# Nap1 stimulates homologous recombination by RAD51 and RAD54 in higher-ordered chromatin containing histone H1

**DOI:** 10.1038/srep04863

**Published:** 2014-05-06

**Authors:** Shinichi Machida, Motoki Takaku, Masae Ikura, Jiying Sun, Hidekazu Suzuki, Wataru Kobayashi, Aiko Kinomura, Akihisa Osakabe, Hiroaki Tachiwana, Yasunori Horikoshi, Atsuhiko Fukuto, Ryo Matsuda, Kiyoe Ura, Satoshi Tashiro, Tsuyoshi Ikura, Hitoshi Kurumizaka

**Affiliations:** 1 Laboratory of Structural Biology, Graduate School of Advanced Science and Engineering, Waseda University, 2-2 Wakamatsu-cho, Shinjuku-ku, Tokyo 162-8480, Japan; 2 Department of Mutagenesis, Division of Chromatin Regulatory Network, Radiation Biology Center, Kyoto University, Yoshidakonoe, Sakyo-ku, Kyoto 606-8501, Japan; 3 Department of Cellular Biology, Research Institute for Radiation Biology and Medicine, Hiroshima University, 1-2-3 Kasumi, Minami-ku, Hiroshima 734-8553, Japan; 4 Division of Gene Therapy Science, Graduate School of Medicine, Osaka University, 2-2 Yamada-oka, Suita, Osaka 565-0871, Japan; 5 These authors contributed equally to this work.

## Abstract

Homologous recombination plays essential roles in mitotic DNA double strand break (DSB) repair and meiotic genetic recombination. In eukaryotes, RAD51 promotes the central homologous-pairing step during homologous recombination, but is not sufficient to overcome the reaction barrier imposed by nucleosomes. RAD54, a member of the ATP-dependent nucleosome remodeling factor family, is required to promote the RAD51-mediated homologous pairing in nucleosomal DNA. In higher eukaryotes, most nucleosomes form higher-ordered chromatin containing the linker histone H1. However, the mechanism by which RAD51/RAD54-mediated homologous pairing occurs in higher-ordered chromatin has not been elucidated. In this study, we found that a histone chaperone, Nap1, accumulates on DSB sites in human cells, and DSB repair is substantially decreased in Nap1-knockdown cells. We determined that Nap1 binds to RAD54, enhances the RAD54-mediated nucleosome remodeling by evicting histone H1, and eventually stimulates the RAD51-mediated homologous pairing in higher-ordered chromatin containing histone H1.

Homologous recombination functions in mitotic DNA double strand break (DSB) repair and meiotic chromosome segregation^1^,^2^. In eukaryotes, RAD51, which is synonymous to RAD51A in human cells, plays a central role in promoting homologous pairing during homologous recombination^1^,^2^. In the initial stage of homologous recombination, RAD51 accumulates on the single-stranded DNA (ssDNA) regions produced on the DSB sites, and forms a helical RAD51-ssDNA nucleoprotein complex with about six RAD51 molecules bound per helical turn. This RAD51-ssDNA complex binds to intact double-stranded DNA (dsDNA), and the homologous sequences between ssDNA and dsDNA are aligned within the RAD51-ssDNA-dsDNA complex (ternary complex). In the ternary complex, ssDNA invades the dsDNA, and a heteroduplex between the ssDNA and the complementary strand of dsDNA is formed (homologous pairing).

In eukaryotes, RAD51 itself is not sufficient to promote homologous pairing in chromosomes, because the wrapping of DNA into nucleosomes in chromatin is inhibitory^3^,^4^. RAD54, a member of the ATP-dependent nucleosome remodeling factor family, is required to overcome the nucleosome barrier for RAD51-mediated homologous pairing^5^,^6^,^7^,^8^,^9^. However, RAD54 is still not sufficient to promote the RAD51-mediated homologous pairing in higher-ordered chromatin, in which linker histones and/or architectural chromatin proteins (such as heterochromatin proteins) are bound to nucleosomes^10^.

In eukaryotes, most nucleosomes bind the linker histone H1, and form the “chromatosome”, which is a basic unit of higher-ordered chromatin^11^,^12^,^13^,^14^. However, the means by which RAD51 and RAD54 function together, to promote homologous pairing in chromatin containing histone H1, have not been elucidated. In the budding yeast *Saccharomyces cerevisiae*, the linker histone H1 homologue, Hho1, suppresses homologous recombination^15^. The histone H1-mediated suppression of DNA repair also occurs in mouse cells^16^. Consistently, the H1.2-deficient mice lacking a major histone H1 variant, H1.2, exhibit increased resistance to apoptosis induced by DSBs^17^. Therefore, histone H1 may generally function as a negative regulator to suppress inappropriate DNA repair by recombination, which may cause chromosomal aberrations.

In the DNA damage response process, chromatin assembly and disassembly may be promoted by the combined actions of an ATP-dependent nucleosome remodeler and histone chaperones^3^,^4^. Histone chaperones catalyze the histone assembly and disassembly reactions, and function in chromatin dynamics^18^,^19^. A recent study demonstrated that the Nap1-family proteins, which are prominent histone chaperones, are required for somatic homologous recombination in *Arabidopsis thaliana*^20^. This strongly suggested that Nap1 may function to alter chromatin structure during homologous recombination.

In the present study, we found that Nap1 accumulates on DSB sites, and may function in the DSB repair probably through homologous recombination in human cells. We evaluated the effects of Nap1 on the homologous recombination reaction in model chromatin templates. In contrast to general expectations, Nap1 did not affect the RAD51/RAD54-mediated homologous pairing in nucleosomal DNA. Surprisingly, however, we found that Nap1 bound to RAD54, and significantly stimulated the RAD51/RAD54-mediated homologous pairing in chromatin containing the linker histone H1, in addition to the core histones. Our results provide direct molecular evidence for the cooperative actions between the nucleosome remodeling factor and the histone chaperone in higher-ordered chromatin to maintain genome integrity.

## Results

### Nap1 functions in DNA double strand break repair by homologous recombination in human cells

**Figure 1:**
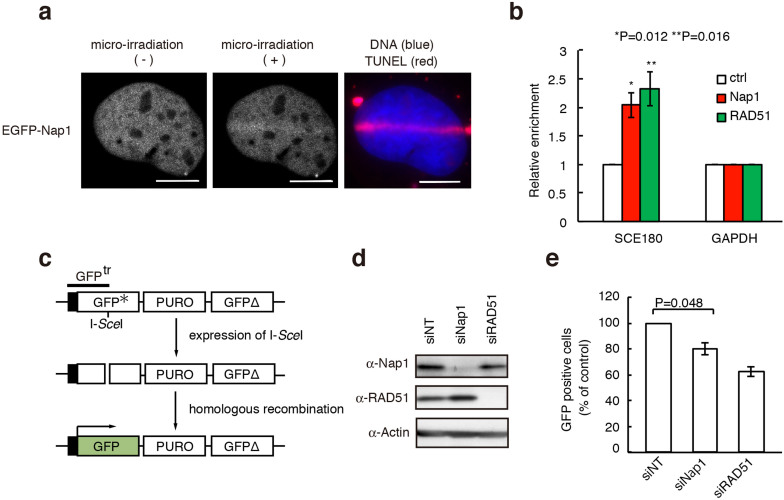
Nap1 accumulates at DSB sites and functions in homologous recombination. (a) Time-lapse analysis of EGFP-NLS-Nap1-expressing GM0637 cells, after laser UVA microirradiation. The EGFP signal was examined before (left panel) and at 3 min (center panel) after microirradiation. After the time-lapse analysis, the cells were fixed and TUNEL staining was performed to detect the DSBs induced by microirradiation (right panel). DNA and TUNEL signals are shown in blue and red, respectively. Scale bars, 10 μm. (b) Detection of Nap1 and RAD51 accumulation around DSB sites by ChIP. Eight hours after transfection of the I-*Sce*I plasmid (pCBASce) into U2OS DR-GFP cells, the accumulation of Nap1 and RAD51 around the DSB was analyzed by ChIP analyses, using specific antibodies. The amounts of immunoprecipitated DNAs from DSB-induced cells were compared with those from cells lacking DSBs, and the relative ratio of immunoprecipitated DNA increased by DSBs is represented. All ChIP analyses were repeated four times. In each experiment, the quantitative PCR reactions were performed twice. Values represent the mean ± SE. SCE180 indicates 180 bp from the DSB site. Ctrl = signal obtained with normal rabbit IgG. (c) The homologous recombination assay in human cells. The reporter containing the GFP* gene (mutant GFP) and the downstream GFP coding sequence (GFPΔ) was inserted into the HeLa cell chromosome (HeLa-DR-GFP cells). Since the GFP* gene contains the I-*Sce*I site within the coding region, it is inactive before the homologous recombination repair. The I-*Sce*I site is cleaved by exogenously expressed I-*Sce*I, and the homologous recombination between GFP* and the downstream GFPΔ sequences is induced. As a consequence of successful homologous recombination, functional GFP is produced, and the green fluorescent signal can be monitored. (d) Expression of Nap1 and RAD51 proteins in the knockdown cells. Nap1 and RAD51 were detected in the parental, Nap1-knockdown, and RAD51-knockdown cells by western blotting. siNT indicates a control siRNA. RAD51 and actin were electrophoresed by 10% SDS-PAGE. Nap1 was electrophoresed by 8% SDS-PAGE. Full images are presented in Supplementary Fig. S9. (e) The percentages of GFP-positive cells from the parental, Nap1-knockdown and RAD51-knockdown cells. Averages of three independent experiments are shown with the SD values.

The Nap1-family proteins are reportedly required for somatic homologous recombination in *Arabidopsis thaliana*^20^. Since somatic homologous recombination mainly occurs during the DSB repair process, we induced DSBs by laser ablation in human cells. We found that Nap1 rapidly accumulated on the DSB sites after DSB induction on a similar time scale to a DNA damage responsive factor, γH2A.X (Fig. 1a and Supplementary Fig. S1a). The accumulation of Nap1 concomitantly with RAD51 at the DSB site was also observed by a chromatin immunoprecipitation (ChIP) assay (Fig. 1b). In the ChIP assay, the DSB was introduced by the exogenously expressed I-*Sce*I endonuclease (Supplementary Fig. S1b).

To test whether the Nap1 knockdown affects homologous recombination in cells, we employed the GFP reporter system^21^,^22^. In this system, the homologous recombination rate was measured as the GFP signal, which was generated by homologous recombination between the mutant inactive GFP reporter gene and the wild type GFP coding sequence. The mutant GFP gene contained an I-*Sce*I cleavage site, and the homologous recombination was initiated by the introduction of the DSB with the exogenously expressed I-*Sce*I endonuclease (Fig. 1c). The mutant GFP sequence was replaced by the wild type GFP sequence, if homologous recombination properly occurred, and the resulting GFP signals were quantified by flow cytometry (Fig. 1c).

We tested the homologous recombination efficiency in the Nap1-knockdown cells. The Nap1 knockdown did not affect the production of the I-*Sce*I and GFP^tr^ (the product from the mutant GFP gene, containing an I-*Sce*I cleavage site) proteins (Supplementary Fig. S1c), and the DSB induction efficiency by I-*Sce*I was minimally affected in the Nap1 knockdown cells (Supplementary Fig. S1d). In the experimental conditions used in this study, the RAD51-knockdown cells exhibited about a 40% reduction in homologous recombination (Fig. 1d and 1e), because the RAD51-independent pathways (or the remaining RAD51) are still active for DSB repair in the cells. We detected a substantial reduction (about 20%) in homologous recombination in the Nap1-knockdown cells (Fig. 1d and 1e). However, this may be an underestimation, because multiple histone chaperones including Nap1-family proteins, which may redundantly function in homologous recombination, exist in human cells^23^,^24^,^25^,^26^,^27^,^28^. Therefore, in human cells, Nap1 may function in the DSB repair pathway, probably through homologous recombination.

### Nap1 binds to RAD54

**Figure 2:**
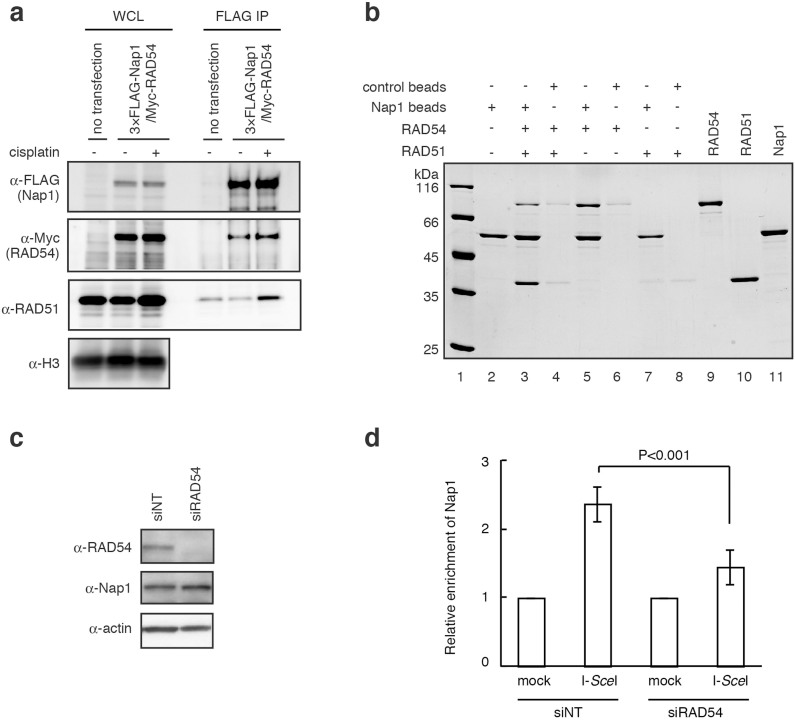
Nap1 interacts with RAD54. (a) Protein-protein interactions in human cells. FLAG-Nap1 was captured with an anti-FLAG antibody. The Myc-RAD54 and endogenous RAD51 that copelleted with FLAG-Nap1 were detected by anti-Myc and anti-RAD51 antibodies, respectively. Similar results were obtained in at least three independent experiments. The gels have been run under the same experimental conditions, and full images are presented in Supplementary Fig. S9. (b) Purified RAD51 and RAD54 were captured by Nap1-conjugated beads. Proteins were detected by SDS-PAGE with Coomassie Brilliant Blue staining. Similar results were obtained in at least three independent experiments. (c) Expression of RAD54 and Nap1 proteins in the RAD54-knockdown cells. RAD54 and Nap1 were detected by western blotting. siNT indicates a control siRNA. Full images are presented in Supplementary Fig. S9. (d) Detection of Nap1 accumulation around DSB sites in the RAD54-knockdown cells. The HeLa DG-GFP cells were transfected with siRAD54 (siRAD54-2), and the DSBs were induced by adenoviruses expressing I-*Sce*I. The accumulation of Nap1 around DSBs was assessed by ChIP analyses, using a specific antibody. The relative immunoprecipitation value represents the ratio of immunoprecipitated DNA after I-*Sce*I digestion, and the Nap1 enrichment on the DSB site was plotted. All ChIP analyses were repeated three times. In each experiment, the quantitative PCR reactions were performed twice. Values represent the mean ± SE.

Nap1 is an acidic histone chaperone, and often functions together with nucleosome remodelers. We then tested whether Nap1 interacts with RAD54, which is the essential nucleosome remodeler for homologous recombination^5^,^6^,^7^,^8^,^9^. Our pull-down assay with exogenously expressed Nap1 and RAD54 proteins in HEK293T cells revealed that Nap1 interacted with RAD54, irrespective of the DNA damage induced by cisplatin (Fig. 2a). We also detected endogenous RAD51 in the Nap1-bound fraction, when the endogenous RAD51 expression was enhanced by cisplatin (Fig. 2a). Interestingly, purified Nap1 did not directly bind to RAD51 (Fig. 2b, lane 7), although it bound to RAD54 (Fig. 2b, lane 5). Purified Nap1 and RAD51 co-pelleted in the presence of RAD54 (Fig. 2b, lane 3), indicating that RAD54 mediates the interaction between Nap1 and RAD51. Consistently, the direct interaction between human RAD51 and RAD54 was reported^29^. Furthermore, Nap1 accumulation at the DSB sites was observed within 3 min (Fig. 1a). RAD54 is also very mobile in cells^30^, suggesting that Nap1 and RAD54 could accumulate at DSB sites on similar time scales. Therefore, Nap1 may be targeted to the DSB sites through its interaction with RAD54.

To test whether the Nap1-RAD54 interaction actually functions in the Nap1 recruitment on the DSB site in human cells, we performed the ChIP experiments in the RAD54 knockdown cells. The DSBs were induced by I-*Sce*I, and the amounts of Nap1 on the DSB sites were assessed in the presence of siRNAs for RAD54 (siRAD54) or control (siNT) (Fig. 2c). The Nap1 accumulation at the DSB sites was reproducibly observed in the presence of the control siNT (Fig. 2d). Interestingly, the Nap1 accumulation was not detected in the presence of the siRAD54, suggesting that RAD54 depletion inhibited the Nap1 recruitment at the DSB sites (Fig. 2d). These results were quite reproducible with a different siRAD54 sequence (Supplementary Fig. S2). Therefore, these results further support the idea that RAD54 mediates Nap1 accumulation on DSB sites in human cells.

### Nap1 may not regulate nucleosome dynamics during homologous pairing

**Figure 3:**
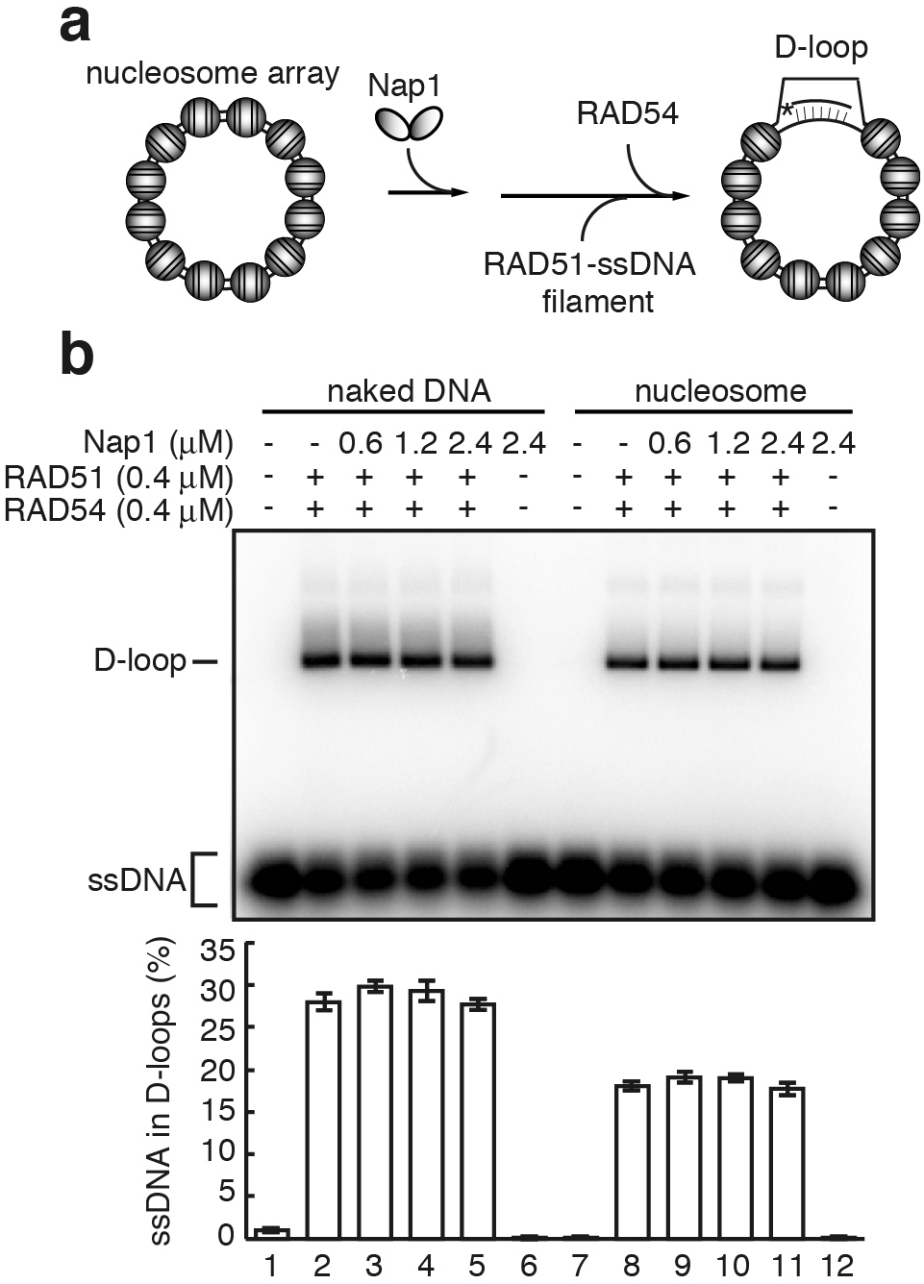
Nap1 does not affect homologous pairing in the nucleosome array. (a) Schematic diagram of the homologous-pairing assay in the nucleosome array. (b) The homologous-pairing assay with either the nucleosome array or naked dsDNA. The indicated amount of Nap1 was added, and the reactions were initiated by the addition of the RAD51-ssDNA (90-mer) filament and RAD54. The lower panel indicates a graphic representation of the experiments shown in the upper panel. The amounts of ssDNA incorporated into the D-loops were quantitated, and the average values of three independent experiments are shown with the SD values.

To analyze the functions of Nap1 in homologous recombination, we performed the *in vitro* homologous recombination assay with reconstituted chromatin (Fig. 3a). We prepared the human core histones H2A, H2B, H3, and H4 as recombinant proteins (Supplementary Fig. S3a)^31^. The nucleosome array was reconstituted with these human core histones by the salt dialysis method. This nucleosome array contains twelve positioned nucleosomes: two nucleosomes with the E4 promoter sequence (E4 di-nucleosome) are located at the center of the nucleosome array, with five specifically positioned nucleosomes formed on the 5*S* rDNA (5*S* nucleosome) flanking both sides of the E4 di-nucleosome^8^,^9^ (Supplementary Fig. S3b). In this nucleosome array, *Eco*RI restriction sites are located in the linker DNA regions flanking each 5*S* rDNA repeat (Supplementary Fig. S3b). Therefore, the nucleosome occupancy on the 5*S* rDNA sequences can be quantified by native gel electrophoresis with the *Eco*RI-treated nucleosome array^9^ (Supplementary Fig. S3c). Alternatively, the nucleosome occupancy on the E4 promoter sequences can be quantified by *Hha*I-treatment of the nucleosome array^8^ (Supplementary Fig. S3b and S3d). In this study, we used a nucleosome array in which the nucleosome occupancies on the 5*S* rDNA and E4 promoter sequences were about 80% and 75%, respectively (Supplementary Fig. S3c and S3d). To determine whether the nucleosome array existed in the soluble fraction, we performed a centrifugation assay. In this assay, the nucleosome array sample without histone H1 (20 μl) was centrifuged, and the top and bottom fractions (10 μl each) were analyzed. Equal amounts of the nucleosomal DNA were detected in both the top and bottom fractions (data not shown), indicating that the nucleosome array remained soluble, and was not aggregated.

We then tested the RAD51-mediated homologous pairing by the D-loop formation assay, using the nucleosome array. In this assay, a ^32^P-labeled ssDNA 90-mer, containing the 5*S* rDNA sequence, was paired with the homologous regions of dsDNA in the nucleosome array, and the D-loops were detected as the product of the homologous-pairing reaction (Fig. 3a). We then tested the effect of Nap1 on the RAD51/RAD54-mediated homologous pairing at the 5*S* rDNA sequences (Fig. 3a). Surprisingly, Nap1 did not affect the RAD51/RAD54-mediated homologous pairing in both naked DNA and nucleosome array templates (Fig. 3b). These results suggested that Nap1 may not function to regulate nucleosome assembly and disassembly during homologous recombination, in contrast to previous expectations.

### Nap1 evicts linker histone H1, and relieves the H1-nucleosome barrier for homologous pairing by RAD51 and RAD54

**Figure 4:**
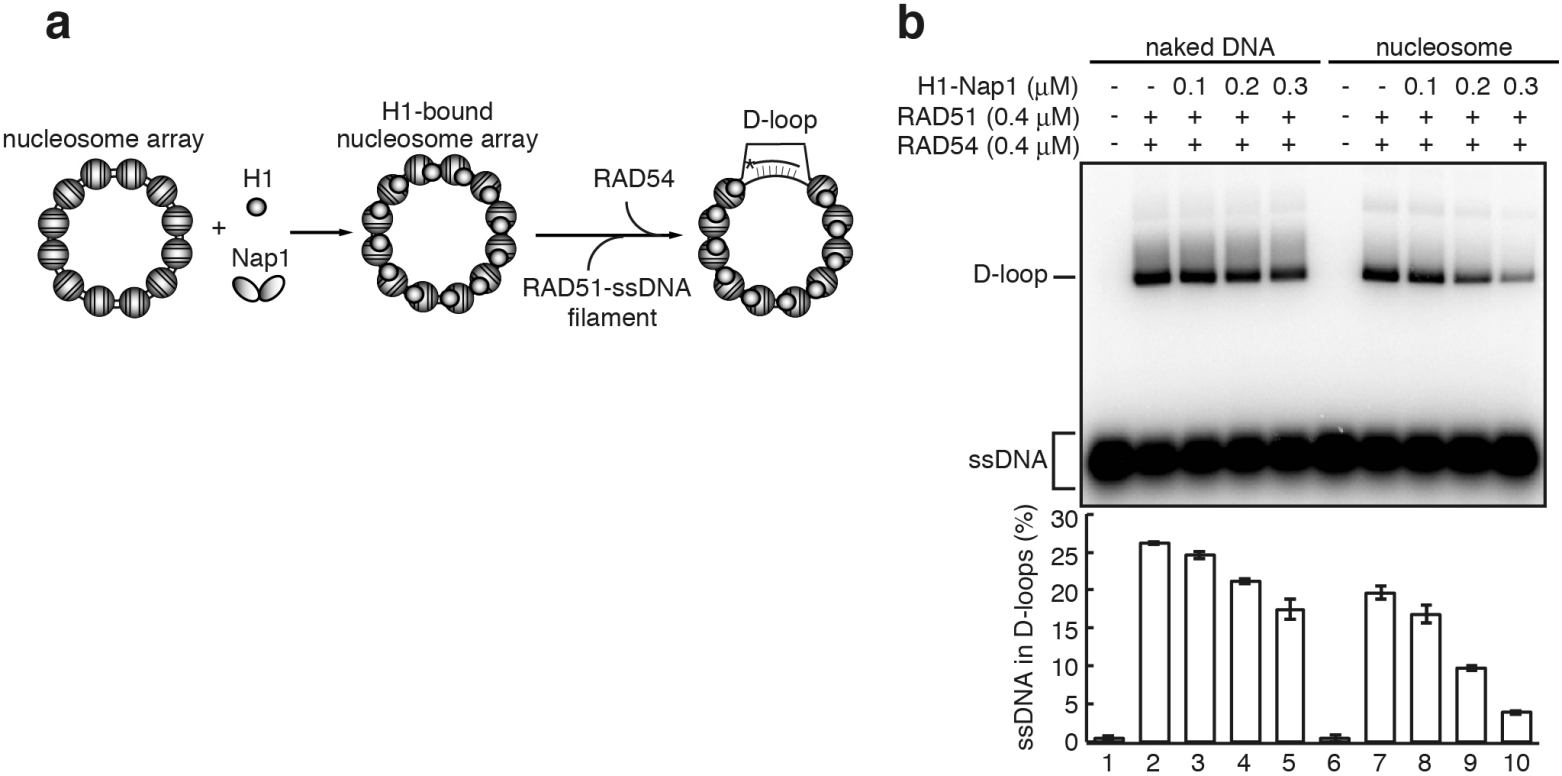
Linker histone H1 suppresses homologous pairing in the nucleosome array. (a) Schematic diagram of the homologous-pairing assay in the nucleosome array with histone H1. (b) The homologous-pairing assay with either the nucleosome array or naked dsDNA in the presence of histone H1. Histone H1 (0.1, 0.2, and 0.3 μM) and Nap1 (Nap1/H1 ratio = 0.5) were added, and the reactions were initiated by the addition of the RAD51-ssDNA (90-mer) filament and RAD54. The lower panel indicates a graphic representation of the experiments shown in the upper panel. The amounts of ssDNA incorporated into the D-loops were quantitated, and the average values of three independent experiments are shown with the SD values.

Therefore, we analyzed the functions of Nap1 in homologous recombination in higher-ordered chromatin, containing the linker histone H1. The human linker histone H1.2 was bacterially expressed, and purified to near homogeneity (Supplementary Fig. S3a). Eleven subtypes of human histone H1 have been identified^32^. We selected human histone H1.2 as a representative linker histone, because H1.2 is ubiquitously and highly expressed in numerous types of cells^32^. We then tested the effect of the linker histone H1 on the RAD51/RAD54-mediated homologous pairing. Nap1 is reportedly required for the proper assembly of the linker histone H1 in chromatin^13^,^33^,^34^. A Nap1/H1 ratio of 0.5 (the ratio is one Nap1 monomer per two H1 monomers) promoted efficient histone H1 binding to nucleosomes, and no H1-free nucleosomes were detected under these conditions (Supplementary Fig. S4a). Therefore, in the present study, the Nap1/H1 ratio of 0.5 was employed as the standard experimental conditions for the H1 assembly onto nucleosome arrays (Fig. 4a).

We subsequently found that histone H1 binding to the nucleosomal DNA template significantly suppressed the RAD51/RAD54-mediated homologous pairing (Fig. 4a and 4b, lanes 6–10). The homologous-pairing suppression by histone H1 was also observed with the naked DNA template, but it was not significant (Fig. 4b, lanes 1–5). To eliminate possibility that excess free histones affected the homologous-pairing reaction, we repeated the experiments in the presence of a heterologous competitor DNA, as a scavenger for excess free histones, and confirmed that the histone H1 loading by Nap1 efficiently suppressed the RAD51/RAD54-mediated homologous pairing (Supplementary Fig. S5, lanes 2, 6, and 10). Micrococcal nuclease (MNase) treatment assays revealed that only trace amounts of nucleosomes without histone H1 were detected under the conditions used in this assay (Supplementary Fig. S4b), and thus most of the nucleosomes in this array were bound to histone H1. The addition of histone H1 without Nap1 significantly reduced the accessibility of MNase to the nucleosomal DNA template, probably by inappropriate H1 binding (Supplementary Fig. S4c). To further confirm the histone H1 binding to nucleosomes, we prepared the nucleosome array containing the biotinylated histone H2A, and evaluated the Nap1-mediated H1 binding to the nucleosome array by the pull-down assay with the streptavidin-conjugated beads. Consistent with the results from the MNase treatment assays, the stoichiometric amount of H1 was detected in the fraction bound to the nucleosome array (Supplementary Fig. S4d and S4e). Nap1 scarcely bound to the nucleosome array, suggesting that Nap1 actually functions as chaperone to promote proper histone H1 assembly on chromatin (Supplementary Fig. S4e). Therefore, we concluded that the proper loading of histone H1 onto the nucleosome array significantly suppresses the RAD51/RAD54-mediated homologous pairing in chromatin.

**Figure 5:**
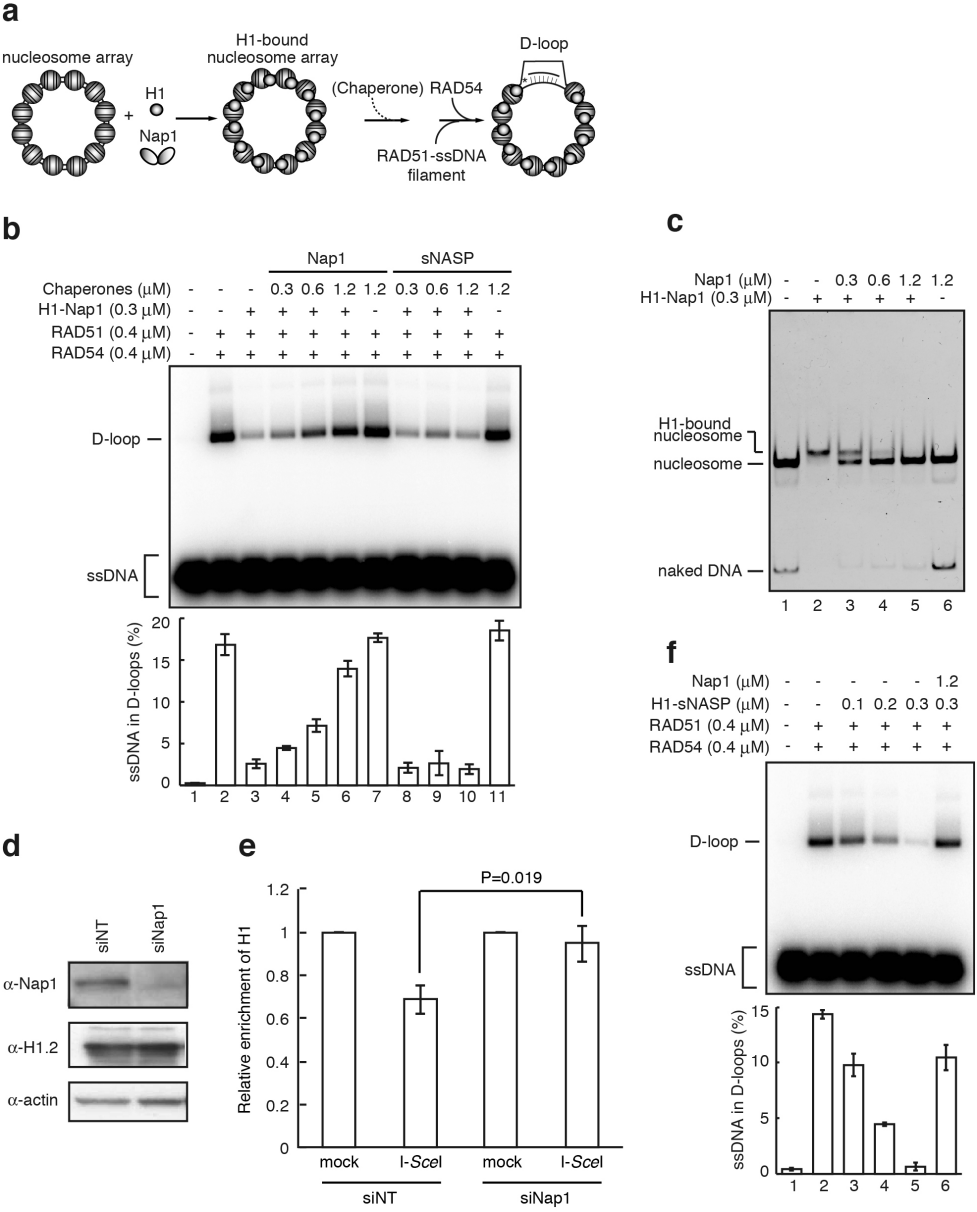
Nap1 relieves the H1-mediated suppression of homologous pairing in the nucleosome array. (a) Schematic diagram. (b) The homologous-pairing assay with a chromatosome array containing histone H1. Histone H1 (0.3 μM) with Nap1 (Nap1/H1 ratio = 0.5) and the indicated amounts of histone chaperone Nap1 or sNASP were added, and the reactions were initiated by the addition of the RAD51-ssDNA (90-mer) filament and RAD54. The lower panel indicates a graphic representation of the experiments shown in the upper panel. The average values of three independent experiments are shown with the SD values. (c) Nap1 titration experiments with chromatosomes reconstituted with a 193 base-pair DNA fragment. Similar results were obtained in at least two independent experiments. (d) Expression of Nap1 and histone H1 proteins in the Nap1-knockdown cells. Nap1 and histone H1 were detected by western blotting. siNT indicates a control siRNA. Full images are presented in Supplementary Fig. S9. (e) Detection of histone H1 around DSB sites in the Nap1-knockdown cells. U2OS DG-GFP cells were transfected with siNap1, and DSBs were induced by pCBASce (I-*Sce*I plasmid). The amounts of histone H1 around the DSBs were analyzed by ChIP analyses, using a specific antibody. The relative immunoprecipitation value represents the ratio of immunoprecipitated DNA after I-*Sce*I digestion, and the histone H1 enrichment on the DSB site was plotted. All ChIP analyses were repeated four times. In each experiment, the quantitative PCR reactions were performed twice. Values represent the mean ± SE. (f) The histone H1-mediated homologous pairing suppression induced by sNASP. Histone H1 (0.1, 0.2, and 0.3 μM) with sNASP (sNASP/H1 ratio = 0.5) was added, in the absence or presence of Nap1 (1.2 μM), and the reactions were initiated by the addition of the RAD51-ssDNA (90-mer) filament and RAD54. The lower panel indicates a graphic representation of the experiments shown in the upper panel. The amounts of ssDNA incorporated into the D-loops were quantitated, and the average values of three independent experiments are shown with the SD values.

We thus performed the Nap1 titration experiments in the homologous-pairing assay, using a nucleosome array containing histone H1 (Fig. 5a). The binding of histone H1 reproducibly and significantly inhibited the RAD51/RAD54-mediated homologous pairing in the nucleosome array (Fig. 5b, lane 3). Interestingly, the homologous-pairing suppression by histone H1 was markedly relieved when the Nap1 concentration was increased (Fig. 5b, lanes 4–6). This result was reproducibly obtained in the presence of a heterologous competitor DNA (Supplementary Fig. S5, lanes 3, 7, and 11). Importantly, another histone H1 chaperone, sNASP^35^,^36^,^37^, did not relieve this histone H1-mediated suppression of homologous pairing (Fig. 5b, lanes 8–10). Consistently, the additional Nap1 actually dissociated histone H1 from the nucleosomes (Fig. 5c). Therefore, when Nap1 is present in amounts greater than the Nap1/H1 ratio of 0.5, it may function specifically to evict histone H1 and to promote homologous pairing in higher-ordered chromatin. Consistent with these *in vitro* results, in cells, the amount of histone H1 was significantly decreased at the I-*Sce*I-induced DSB site, probably by the Nap1-mediated H1 eviction, and this histone H1 eviction was not observed in the Nap1-knockdown cells (Fig. 5d and e).

Intriguingly, as with Nap1, suppression of the Rad51/RAD54-mediated homologous pairing was also observed with histone H1 assembled on the nucleosomal DNA template by sNASP (Fig. 5f, lanes 3–5). In addition, Nap1 relieved the histone H1-mediated homologous pairing suppression induced by sNASP (Fig. 5f, lane 6).

### Nap1 relieves the H1-mediated suppression of nucleosome remodeling by RAD54

**Figure 6:**
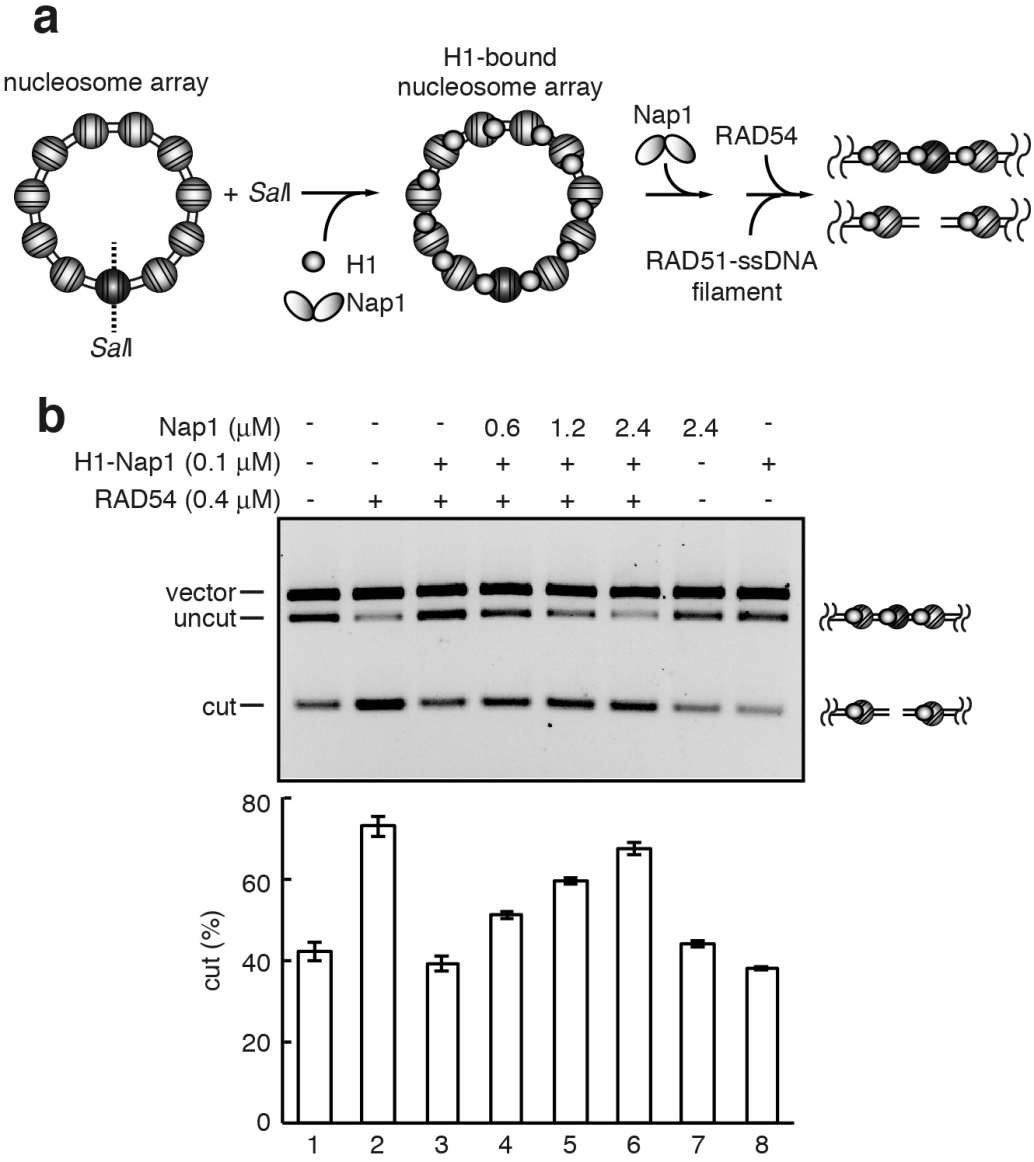
Nucleosome remodeling in chromatosomes. (a) Schematic diagram of the nucleosome remodeling assay. (b) The nucleosome remodeling assay. Histone H1 (0.1 μM) with Nap1 (Nap1/H1 ratio = 0.5) and Nap1 (0.6, 1.2, and 2.4 μM) were added, and the reactions were initiated by the addition of the RAD51-ssDNA (70-mer) filament and RAD54 in the presence of *Sal*I. The lower panel indicates a graphic representation of the experiments shown in the upper panel. The average values of three independent experiments are shown with the SD values.

We next tested the nucleosome remodeling by RAD54. To do so, we reconstituted nucleosome arrays formed on eleven 5*S* rDNAs. In these 5*S* rDNA nucleosome arrays, a single *Sal*I restriction site is located within the central nucleosome (the nucleosome occupancy was about 65–70%, as estimated by the *Sal*I cleavage for 10–60 min) (Fig. 6a and Supplementary Fig. S6). The remodeling of the central nucleosome containing the *Sal*I site can be monitored by the enhanced or decreased accessibility of the *Sal*I nuclease to the nucleosomal DNA^38^. Since the RAD54-mediated nucleosome remodeling is reportedly enhanced by the RAD51-ssDNA complex^8^, we performed the nucleosome remodeling assay in the presence of the RAD51-ssDNA complex. As shown in Fig. 6b, the *Sal*I cleavage that was inhibited by nucleosome formation (lane 1) was significantly enhanced upon nucleosome remodeling by RAD54 (lane 2). When histone H1 was assembled onto the nucleosome array, the *Sal*I cleavage was inhibited (Fig. 6b, lane 3), indicating that histone H1 binding restricts the nucleosome remodeling by RAD54. Interestingly, the addition of Nap1 significantly relieved the suppression of the RAD54-mediated nucleosome remodeling, in a Nap1 concentration-dependent manner (Fig. 6b, lanes 4–6). Nap1 alone did not affect the *Sal*I cleavage efficiency in the nucleosome array (Fig. 6b, lane 7). Therefore, we concluded that, during the homologous pairing processes, Nap1 evicts histone H1 from chromatin, enhances RAD54-mediated nucleosome remodeling, and eventually activates RAD51-mediated homologous pairing within chromosomes.

### The specific Nap1-RAD54 Interaction is required for relieving the H1-mediated suppression of homologous pairing and nucleosome remodeling

**Figure 7:**
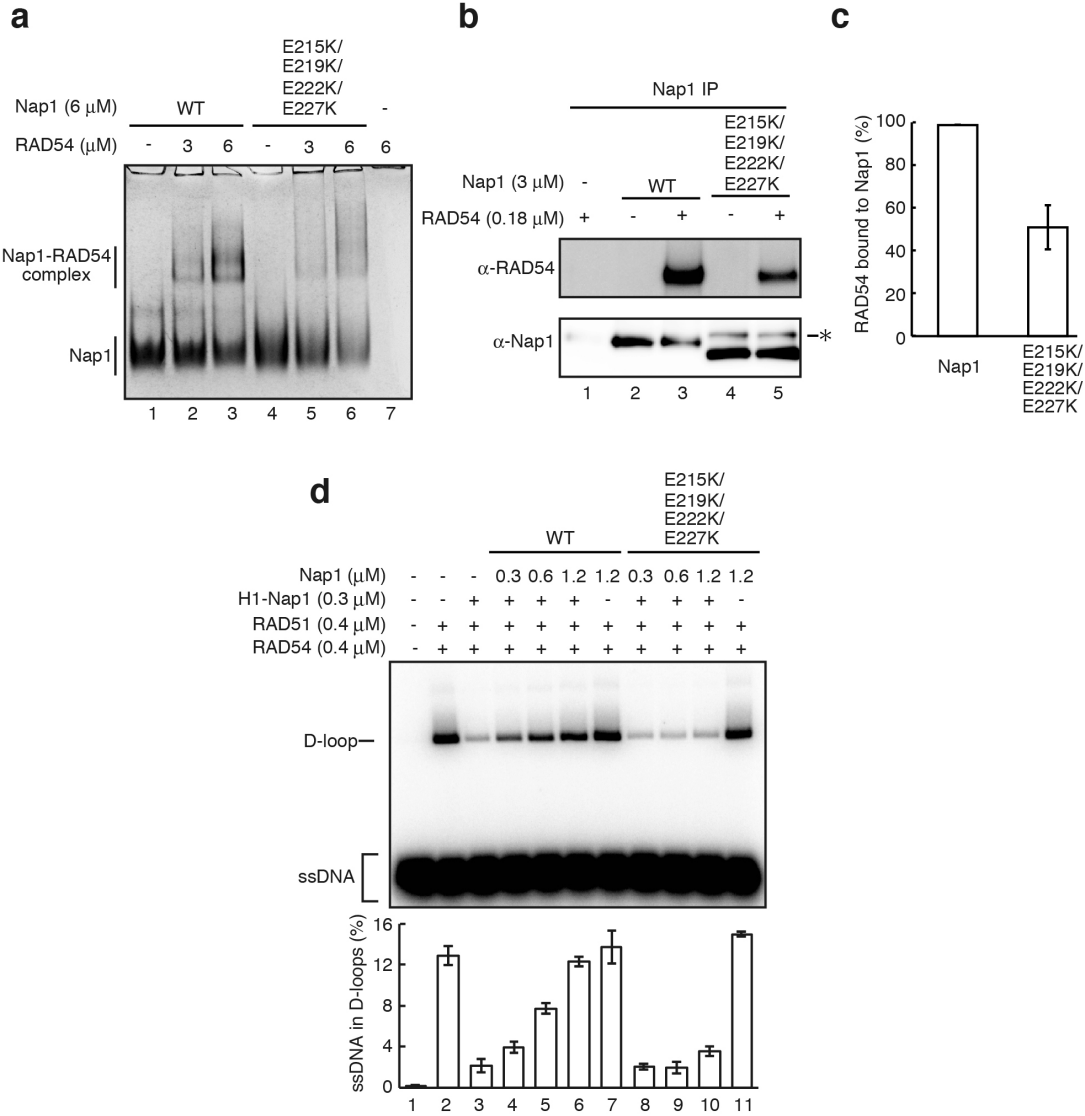
The Nap1-RAD54 interaction is required to relieve the H1-mediated suppression of homologous recombination in higher-ordered chromatin. (a) The gel electrophoretic mobility shift assay. Wild type Nap1 or the Nap1(E215K,E219K,E222K,E227K) mutant was incubated with the indicated amounts of RAD54, and the Nap1-RAD54 complexes were analyzed by 5% native PAGE with Coomassie Brilliant Blue staining. In this condition, RAD54 did not enter the gel (lane 7), because of its basic pI. Similar results were obtained in at least three independent experiments, performed under different conditions. (b) The pull-down assay. Purified RAD54 was incubated with Nap1 or the Nap1(E215K,E219K,E222K,E227K) mutant, and the RAD54 bound to Nap1 or the Nap1(E215K,E219K,E222K,E227K) mutant was captured by the anti-Nap1antibody. The RAD54 bound to Nap1 or the Nap1(E215K,E219K,E222K,E227K) mutant was detected by an anti-RAD54 antibody (upper panel). Nap1 and the Nap1(E215K,E219K,E222K,E227K) mutant were detected by an anti-Nap1 antibody (lower panel). The asterisk indicates the read-through product. The gels were run under the same experimental conditions, and full images are presented in Supplementary Fig. S9. (c) Graphic representation of the experiments shown in (b). The average values of three independent experiments are shown with the SD values. (d) The homologous-pairing assay with a chromatosome array containing histone H1. Histone H1 (0.3 μM) with Nap1 (Nap1/H1 ratio = 0.5) and the indicated amounts of Nap1 or the Nap1(E215K,E219K,E222K,E227K) mutant were added, and the reactions were initiated by the addition of the RAD51-ssDNA (90-mer) filament and RAD54. The lower panel indicates a graphic representation of the experiments shown in the upper panel. Amounts of ssDNA incorporated into the D-loops were quantitated, and the average values of three independent experiments are shown with the SD values.

To test whether the Nap1-RAD54 interaction is actually involved in the activation of homologous pairing in higher-ordered chromatin, we purified the Nap1(E215,219,222,227K) mutant, in which the four acidic Glu215, Glu219, Glu222, and Glu227 residues are replaced by basic Lys residues. The Nap1(E215,219,222,227K) mutant migrated slightly faster than wild type Nap1, and a trace amount of the read-through product was present as a contaminant in the purified fraction (Supplementary Fig. S7a). These Nap1 glutamate residues are exposed to the solvent^39^, and potentially interact with basic proteins, such as RAD54 (Supplementary Fig. S8). Our native polyacrylamide gel electrophoretic analysis revealed that the Nap1(E215,219,222,227K) mutant was clearly defective in the complex formation with RAD54 (Fig. 7a). A pull-down assay with an anti-Nap1 antibody also revealed that the Nap1(E215,219,222,227K) mutant was defective in the RAD54 binding (Fig. 7b and 7c).

Interestingly, we found that the Nap1(E215,219,222,227K) mutant was quite defective in relieving the histone H1-mediated homologous pairing suppression (Fig. 7d, lanes 8–10), as compared to wild type Nap1 (Fig. 7d, lanes 4–6). Although the Nap1(E215,219,222,227K) mutant still formed the complex with Nap1 with about 50% efficiency (Fig. 7b and c), it may not properly form the active complex with RAD54, because the bands corresponding to the complex containing the Nap1 mutant and RAD54 were smeared, as compared to those of the Nap1-RAD54 complexes, on the native polyacrylamide gel electrophoretic analysis (Fig. 7a). In contrast, this mutant was completely proficient in the nucleosome assembly and histone H1 eviction activities (Supplementary Fig. S7b and S7c). These results suggested that the Nap1-RAD54 interaction plays an important role in the RAD51/RAD54-mediated homologous pairing in higher-ordered chromatin containing histone H1.

## Discussion

Previous biochemical experiments revealed that RAD51 promotes homologous pairing between ssDNA and naked dsDNA^1^,^2^. However, the genomic DNA is not naked, and is wrapped within nucleosomes, which inhibit DNA processing, including homologous recombination. The yeast, fly, and human RAD51 proteins require a cognate ATP-dependent nucleosome remodeler, RAD54, to promote homologous pairing in nucleosomal dsDNA *in vitro*^5^,^6^,^7^,^8^,^9^. Furthermore, in the nucleus, most of the nucleosomes are associated with the linker histone H1 and form chromatosomes, in which one histone H1 binds to one nucleosome^11^. The formation of higher-ordered chromatin containing histone H1 is generally repressive for homologous recombination in cells^15^,^16^,^17^,^40^. Consistently, we found that histone H1 significantly inhibits the homologous-pairing reaction in chromatin (Fig. 4). Therefore, the elucidation of the mechanism by which RAD51, together with RAD54, promotes homologous pairing in chromatin containing histone H1 is emerging as an important issue to be solved.

The histone chaperone Nap1-family proteins are reportedly required for somatic homologous recombination in *Arabidopsis*, a plant^20^. Consistently, we showed that, in human cells, Nap1 accumulates on the DSB sites, and is required for homologous recombination (Fig. 1). Nap1 is known as a major core histone chaperone^41^,^42^,^43^,^44^,^45^,^46^,^47^,^48^,^49^. Therefore, Nap1 was first considered to function in the nucleosome assembly/disassembly processes during homologous recombination^20^. However, we unexpectedly found that Nap1 does not affect the RAD51/RAD54-mediated nucleosome remodeling and homologous pairing in nucleosomal DNA templates (Fig. 3). This suggested that Nap1 may function in higher-ordered chromatin, but not at the nucleosome level, during homologous recombination. Nap1 reportedly functions as the chaperone for the linker histone H1^13^,^33^,^34^, as well as for the core histones H2A–H2B and H3–H4. These findings encouraged us to test the Nap1 activity in RAD51/RAD54-mediated homologous pairing in chromatin containing histone H1. To our surprise, our results revealed that Nap1 directly binds to RAD54 (Fig. 2), and stimulates the RAD51/RAD54-mediated homologous pairing in chromatin containing histone H1 (Fig. 5). The significance of the Nap1-RAD54 interaction was also suggested by further *in vitro* and *in vivo* experiments. The Nap1(E215,219,222,227K) mutant, which is partially defective in RAD54 binding, did not relieve the H1-mediated suppression of the RAD51/RAD54-mediated homologous pairing in chromatin (Fig. 7). Consistently, the accumulation of Nap1 at the DSB site was significantly reduced in the RAD54-depleted cells (Fig. 2c and 2d).

Linker histones restrict nucleosome mobility, and thus significantly influence the accessibility of DNA binding proteins in higher-ordered chromatin^50^,^51^. In this context, histone H1 may restrict nucleosome mobility, which is required for nucleosome remodeling by RAD54 during the RAD51-mediated homologous pairing. We found that histone H1 inhibits the nucleosome remodeling imposed by RAD54 (Fig. 6). We then showed that Nap1 significantly relieves the H1-dependent suppression of the RAD54-mediated nucleosome remodeling, by evicting histone H1 from chromatin (Figs. 5 and 6). Consistently, the H1 eviction from the DSB site did not occur in the Nap1-depleted cells (Fig. 5d and 5e). Therefore, Nap1 may function to reduce the H1 concentration at the DSB site. Unlike the core histones, histone H1 binds to chromatin and dissociates on a fairly rapid time-scale^52^. The interaction of Nap1 with RAD54 and indirectly with RAD51 might increase the local concentration of Nap1 at the DSB sites, thus increasing the mobility of nucleosomes by facilitating the removal of histone H1 in higher-ordered chromatin.

The histone H1-dependent inhibition of homologous pairing may be important to suppress inappropriate recombination, which may cause chromosomal aberrations^15^,^16^. On the other hand, the H1-mediated recombination suppression must be relieved during the somatic and meiotic homologous recombination reactions, which are essential for the maintenance and accurate inheritance of genetic information. The findings presented here will help to clarify how homologous pairing by RAD51 is accomplished within the chromatosome, which is the major basic unit of higher-ordered chromatin in higher eukaryotes.

## Methods

### Preparation of proteins

Human histone H1.2 was produced in *Escherichia coli* BL21 (DE3) cells, as a His_6_-SUMO-tagged protein. The His_6_-SUMO-tag was proteolytically removed during the purification procedures, and the purified histone H1.2 was stored at −80°C in buffer C. The details for the histone H1.2 purification are described in the Supplementary Methods. Human histones H2A, H2B, H3.1, and H4 were purified by the method described previously^31^. Human Nap1^46^, sNASP^53^, and RAD51^54^ were purified as described previously. The human RAD54 cDNA was inserted into the pFastBac HTc vector (Life Technologies), and the recombinant human RAD54 baculovirus was generated^55^. The details for the RAD54 purification are described in the Supplementary Methods.

### Preparation of DNAs

HPLC-purified oligonucleotides were purchased (Nihon Gene Research Laboratory) for use as the ssDNA substrates in the D-loop formation and nucleosome remodeling assays, and are listed in Supplementary Table S1. The superhelical dsDNAs were prepared as described^56^. To prevent the superhelical dsDNA from undergoing irreversible denaturation, alkaline treatment of the cells harboring the plasmid DNA was avoided. The cells were gently lysed using sarkosyl, as described^56^. A 193-base-pair DNA fragment containing the Widom 601 sequence^57^ was prepared by the method described previously^58^. DNA concentrations are expressed in moles of nucleotides.

### Assembly of nucleosome arrays and nucleosomes

Nucleosome arrays were reconstituted on the plasmid DNAs, as described previously^9^. The details are described in the Supplementary Methods.

### Assay for homologous pairing

RAD51 (400 nM) was incubated with the ^32^P-labeled 5*S* 90-mer single-stranded oligonucleotide (1 μM) at 37°C for 10 min, in the presence of 1 mM MgCl_2_, 1 mM CaCl_2_, and 1 mM ATP. Histone H1 was mixed with Nap1 at a 2:1 ratio, and the sample was kept on ice for 20 min to form the H1-Nap1 complex. The H1-Nap1 complex was added to naked dsDNA or nucleosomal dsDNA. After a 10 min incubation at 37°C, Nap1 or Nap1 storage buffer was added to the H1-bound naked dsDNA or nucleosomal dsDNA, and the solutions were incubated further for 6 min. Subsequently, the RAD51-ssDNA complex (5.5 μl) and RAD54 (400 nM, 1.5 μl) were added to the H1-bound naked dsDNA or nucleosomal dsDNA (30 μM) in 10 μl of reaction buffer, containing 22 mM HEPES-NaOH (pH 7.5), 9 mM Tris-HCl (pH 7.5), 43 mM NaCl, 80 mM KCl, 0.2 mM EDTA, 1 mM DTT, 0.6 mM 2-mercaptoethanol, 15 μM phenylmethylsulfonyl fluoride, 7.5% glycerol, 1 mM MgCl_2_, 1 mM CaCl_2_, 1 mM ATP, 20 mM creatine phosphate, 75 μg/ml creatine kinase, and 100 μg/ml BSA. After a 10 min incubation at 37°C, the reactions were terminated by the addition of 2 μl of stop solution, containing SDS (0.2%) and proteinase K (1.4 mg/ml, Roche Applied Science), and the deproteinized DNA products were separated by 1% agarose gel electrophoresis in 1× TAE buffer at 4 V/cm for 2 hr. The gels were dried, and visualized using an FLA-7000 imaging analyzer (GE Healthcare).

### Assay for nucleosome remodeling

RAD51 (0.4 μM) was incubated with the *Sal*I 70-mer single-stranded oligonucleotide (1 μM) at 37°C for 10 min, in the presence of 1 mM MgCl_2_, 0.5 mM CaCl_2_, and 1 mM ATP. The nucleosome array was reconstituted on the pB5*S*array DNA, and was treated with *Sal*I (7 units; TOYOBO) for 30 min at 30°C. Histone H1 (0.1 μM) was mixed with Nap1 (0.05 μM), and the sample was kept on ice for 20 min. The resulting H1-Nap1 complex was added to the nucleosome array (5 μM). After a 10 min incubation at 30°C, Nap1 or Nap1 storage buffer was added to the chromatosome array, and the samples were incubated for 5 min. Subsequently, RAD54 (0.4 μM, 3 μl), together with the RAD51-ssDNA complex (2 μl), was added to the reaction mixture in 20 μl of reaction buffer, containing 22 mM HEPES-NaOH (pH 7.5), 10 mM Tris-HCl (pH 7.5), 84 mM KCl, 42 mM NaCl, 0.2 mM EDTA, 0.5 mM 2-mercaptoethanol, 1 mM DTT, 20 μM phenylmethylsulfonyl fluoride, 9% glycerol, 2 mM MgCl_2_, 0.05 mM CaCl_2_, 2 mM ATP, and 110 μg/ml BSA. After a 20 min incubation at 30°C, the reactions were terminated by the addition of 4 μl of stop solution, containing SDS (0.2%) and proteinase K (1.4 mg/ml, Roche Applied Science). The DNA was extracted by phenol/chloroform, and was precipitated with ethanol. The resulting DNA samples were cleaved by *Not*I (TOYOBO) and *Hind*III (TOYOBO), and were analyzed by 1.2% agarose gel electrophoresis. The products were visualized by SYBR Gold (Invitrogen) staining.

### Assay for protein-protein interactions

The RAD51- and RAD54-binding or the RAD51-RAD54-binding assays were performed *in vitro* and *in vivo*. The details for the protein-protein interaction assays are described in the Supplementary Methods.

### Time-lapse analysis of EGFP-NLS-Nap1-expressing GM0637 cells after laser UVA microirradiation

GM0637 cells, a simian virus 40-transformed fibroblast cell line, were cultured in Dulbecco's modified Eagle's medium supplemented with 10% fetal calf serum. The GM0637 cells were grown on glass cover slips. The GM0637 cells were transiently transfected with the EGFP-NLS-Nap1 plasmid, using GeneJuice (Novagen), and maintained on the microscope stage in a Chamlide TC live cell chamber system (Live Cell Instrument) at 37°C. The NLS amino acid sequence is Pro-Lys-Lys-Lys-Arg-Lys-Val-Glu. Imaging and microirradiation experiments were performed using an LSM510 confocal laser scanning microscope (Carl Zeiss) with a 40×/1.2C-Apochromat objective. For the induction of DNA damage, GM0637 cells were microirradiated^59^. For sensitization, the cultures were treated for 10 min with 2 μg/ml Hoechst 33258 (Sigma), and then the culture medium was replaced by Leibovitz's L-15 (Gibco), containing 10% FBS and 25 mM HEPES (Gibco). The 364-nm line of the UVA laser was used for microirradiation. The 488 nm Ar laser line was used for imaging. The EGFP signal was examined for 3 min after microirradiation. After the time-lapse analysis, the cells were rinsed with 1 × PBS, and then soaked in cytoskeleton buffer (100 mM NaCl, 300 mM sucrose, 10 mM PIPES (pH 6.8), 3 mM MgCl_2_, 1 mM EGTA, and 0.5% Triton X-100) for 5 min on ice. The cells were washed with cold PBS and fixed with 4% paraformaldehyde in 1× phosphate-buffered saline (PBS) for 10 min at room temperature. TUNEL staining using In situ Cell Death Detection Kits (Roche) was performed to detect the DSBs induced by microirradiation, according to the manufacturer's recommendations.

### Homologous recombination repair assay

The homologous recombination repair assay was performed as previously reported^21^,^22^. In this system, the gene conversion type of homologous recombination occurs, and the intervening PURO sequence is not deleted. Briefly, 2 μg of the I-*Sce*I expression vector (pCBASce) was introduced into HeLa-DR-GFP cells, together with either 5 nM ON-TARGETplus siRNA (Dharmacon) for the non-targeting control (siNT), Nap1 (siNap1), or RAD51 (siRAD51), respectively, using Lipofectamine 2000 as recommended by the manufacturer (Invitrogen). To determine the amount of HR repair, the percentage of GFP-positive cells was quantified by flow cytometry two days after transfection, using a FACSCanto II flow cytometer (Becton Dickinson).

### Chromatin immunoprecipitation assay

The I-*Sce*I protein was expressed in U2OS DR-GFP cells bearing the pCBASce vector introduced by electroporation (BIO-RAD) or in HeLa DR-GFP cells with adenoviruses expressing I-*Sce*I (Invitrogen) by infection. After 8 hr, the cells were treated with formaldehyde (final concentration, 1%) for 10 min at room temperature. For the depletion of Nap1, 10 nM of ON-TARGETplus siRNA (Dharmacon) for Nap1 (siNap1) or the non-targeting control (siNT) was introduced into the U2OS DR-GFP cells at 48 hr before the transfection of pCBASce, using Lipofectamine RNAimax (Invitrogen). For the depletion of RAD54, a Stealth siRNA (5 nM) for RAD54 (siRAD54-1 or siRAD54-2) (Invitrogen) was introduced into U2OS DR-GFP or HeLa cells. The siRAD54-1 and siRAD54-2 sequences are indicated below.

siRAD54-1: 5′-UUGGUUAGCUGACUCAAAGGUUUCC-3′.

siRAD54-2: 5′-AAAUGCUUCAUGCUGACUGCUGUCC-3′.

The cells were then sonicated, and the chromatin suspensions were prepared. Immunoprecipitations were performed using an anti-Nap1 polyclonal antibody, an anti-RAD51 polyclonal antibody, or an anti-histone H1.2 polyclonal antibody (abcam). Normal rabbit IgG was used as a negative control. Real-time PCR reactions were performed using a LightCycler or ABI 7500 PCR System (SYBR *Premix Ex Taq*, TAKARA). The primers used for the detection of the I-*Sce*I break site were a set hybridizing at a distance of 180 bp from the DSB site: SCE180-F (5′-CATGCCCGAAGGCTACGT-3′) and SCE180-R (5′-CGGCGCGGGTCTTGTA-3′). The GAPDH locus was amplified as an internal control for normalization, using the primers GAPDH-F (5′-TCTCCCCACACACATGCACTT-3′) and GAPDH-R (5′-CCTAGTCCCAGGGCTTTGATT-3′). The relative immunoprecipitation value represents the immunoprecipitated DNA ratio after the DSB induction by I-*Sce*I relative to immunoprecipitated DNA after vehicle treatment.

For the quantification of DSBs induced by I-*Sce*I, genomic DNA was extracted using MagExtractor (Genome) (TOYOBO) from the cells under the same conditions as the ChIP analysis. Real-time PCR reactions were performed using a LightCycler PCR instrument (FastStart DNA Master SYBR Green1, Roche). The primers used for the quantification of DSBs covering the I-*Sce*I site were ISCE1-F (5′-TGTCCGGCTAGGGATAACAG-3′) and ISCE1-R (5′- AAGTCGTGCTGCTTCATGTG-3′). The primers GAPDH-F and GAPDH-R were used as a control. The relative DNA value represents the DNA ratio after the DSB induction by I-*Sce*I relative to DNA after vehicle treatment. Genomic DNA was prepared from three independent experiments. All qPCR assays were performed in duplicate. Values represent the mean ± SE.

## Author Contributions

S.M. and M.T. performed most of the biochemical analyses. W.K., H.T. and A.O. purified proteins. S.M., M.I. and T.I. performed the *in vivo* pull-down assays. J.S., H.S., A.K., Y.H., A.F., R.M., T.I. and S.T. performed the *in vivo* assays for protein accumulation and homologous recombination. K.U. provided advice for the histone H1 assembly experiments with Nap1. H.K. conceived, designed, and supervised all of the work, and H.K., S.M. and M.T. wrote the paper. All of the authors discussed the results and commented on the manuscript.

## Supplementary Material

Supplementary Information Supplementary Figures and Methods
