# Supplementary material for: Nap1 stimulates homologous recombination by RAD51 and RAD54 in higher-ordered chromatin containing histone H1
Source: Sci Rep. 2014 May 6;4:4863. doi: 10.1038/srep04863 (PMC4010968; doi:10.1038/srep04863)
Supplement: Supplementary Information — Supplementary Figures and Methods [file srep04863-s1.pdf]

Supplemental Information

Nap1 stimulates homologous recombination by RAD51 and RAD54 in higher-ordered chromatin containing histone H1

Shinichi Machida, Motoki Takaku, Masae Ikura, Jiying Sun,
Hidekazu Suzuki, Wataru Kobayashi, Aiko Kinomura, Akihisa Osakabe,
Hiroaki Tachiwana, Yasunori Horikoshi, Atsuhiko Fukuto, Ryo Matsuda,
Kiyoe Ura, Satoshi Tashiro, Tsuyoshi Ikura, and Hitoshi Kurumizaka

Supplementary Figures S1-S10
Supplementary Table 1
Supplementary Methods
Supplementary Reference

Supplementary Figures and Legends

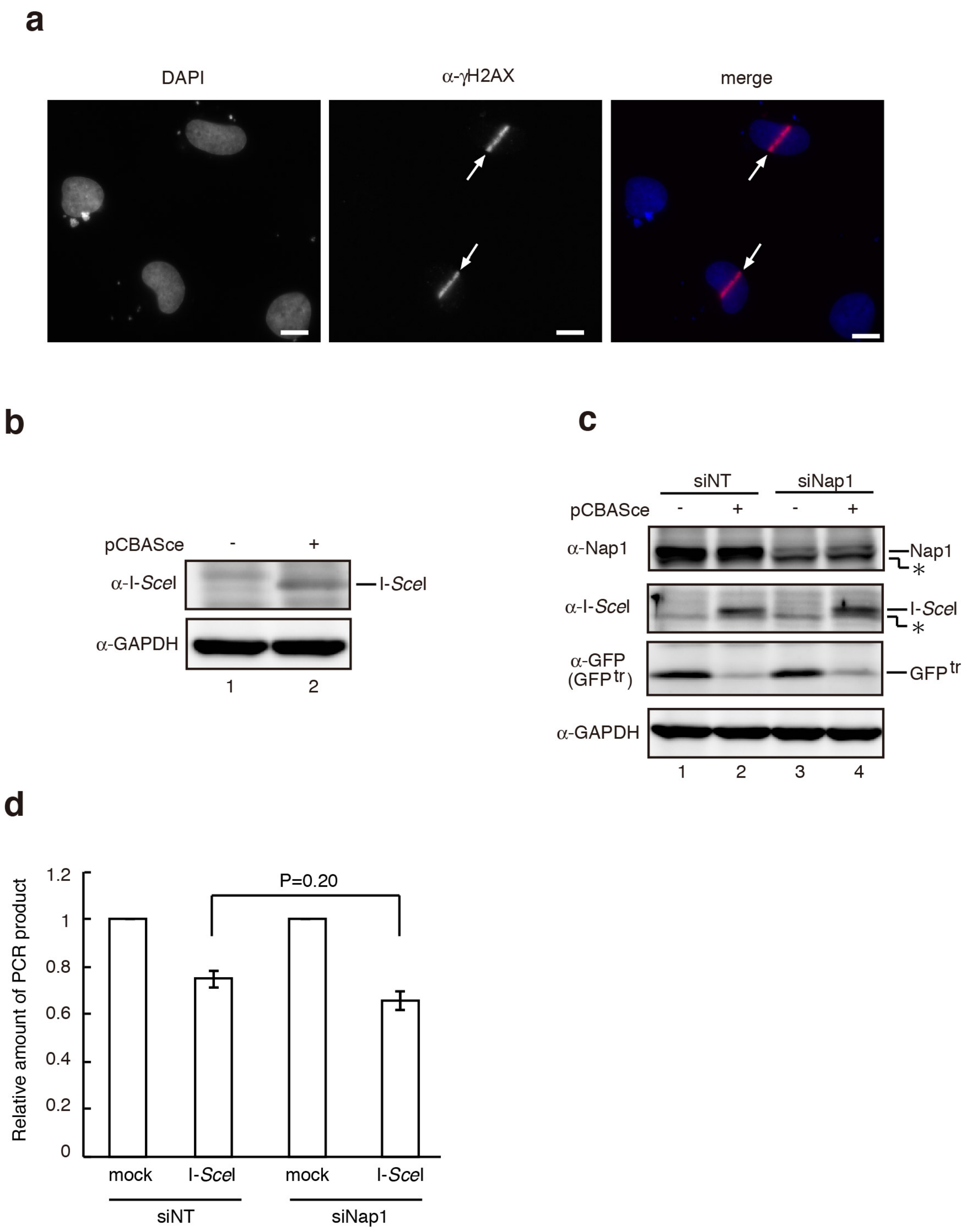

Supplementary Figure S1. Nap1 functions in homologous recombination. (**a**) GM02063 cells were subjected to UVA microirradiation. At 10 min after microirradiation, GM02063 cells were immunostained with an anti-γH2AX

antibody. γH2AX and DAPI signals are shown in red and blue, respectively, in the merged images. Scale bars, 10 µm.

(**b**) Expression of I-*Sce*I in U2OS DR-GFP cells. I-*Sce*I was detected by western blotting with an anti-I-*Sce*I polyclonal antibody (FL-86, Santa Cruz Biotechnology). Full-length blots are presented in Supplementary Fig. S10.

(**c**) Expression of I-*Sce*I and GFP^{tr} in Nap1-knockdown cells. The I-*Sce*I and GFP^{tr} (the product from the mutant GFP gene containing an I-*Sce*I cleavage site) proteins were detected by western blotting with an anti-I-*Sce*I polyclonal antibody (FL-86, Santa Cruz Biotechnology) and an anti-GFP polyclonal antibody (598, MBL). Asterisks indicate non-specific bands. siNT indicates a control siRNA. Full-length blots are presented in Supplementary Fig. S10. (**d**) The DSB formation at the I-*Sce*I site was estimated by PCR. The amounts of the PCR products around the I-*Sce*I site in the I-*Sce*I-induced cells relative to the I-*Sce*I-uninduced cells were plotted. Genomic DNAs extracted from the control (siNT) and Nap1-knockdown (siNap1) cells were analyzed. Genomic DNA was prepared from three independent experiments. All quantitative PCR reactions were performed in duplicate. Values represent the mean ± SE.

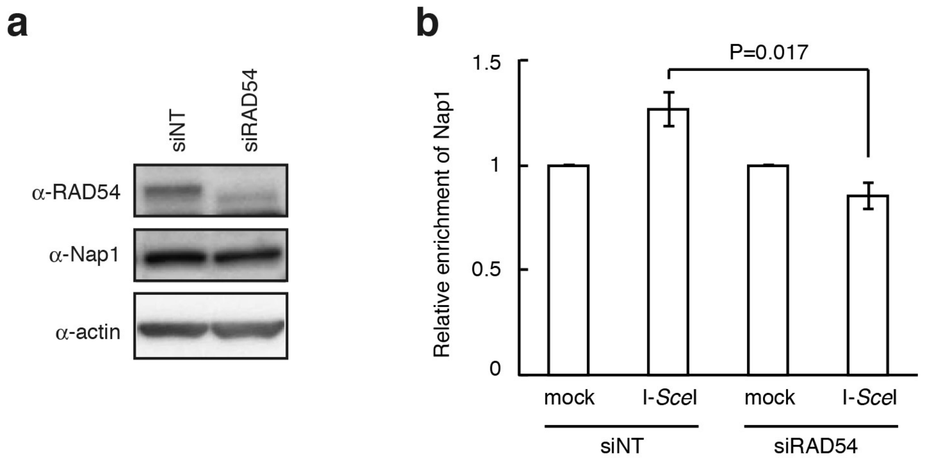

Supplementary Figure S2. Nap1 accumulation around DSB sites in the U2OS DG-GFP cells transfected with siRAD54.

(**a**) Expression of RAD54 and Nap1 proteins in the RAD54-knockdown cells. RAD54 and Nap1 were detected by western blotting. siNT indicates a control siRNA. Full images are presented in Supplementary Fig. S10.

(**b**) Detection of Nap1 accumulation around DSB sites in the RAD54-knockdown cells. The U2OS DG-GFP cells were transfected with siRAD54-1, and the DSBs were induced by exogenously expressed I-*Sce*I. The accumulation of Nap1 around DSBs was analyzed by ChIP analyses, using a specific antibody. The amounts of immunoprecipitated DNAs from DSB-induced cells were compared with those from DSB-uninduced cells, and the relative ratio of Nap1 accumulation was estimated from the amount of immunoprecipitated DNA. All ChIP analyses were repeated three times, and in each experiment, the quantitative PCR reactions were performed in duplicate. Values represent the mean ± SE. The Nap1 accumulation at the DSB site was reproducibly observed in the presence of the control siNT, although the accumulation rate was moderate, as compared to the Nap1 accumulation without siRNA (Fig. 1b). This may be due to the siRNA treatment, which may be toxic for cells.

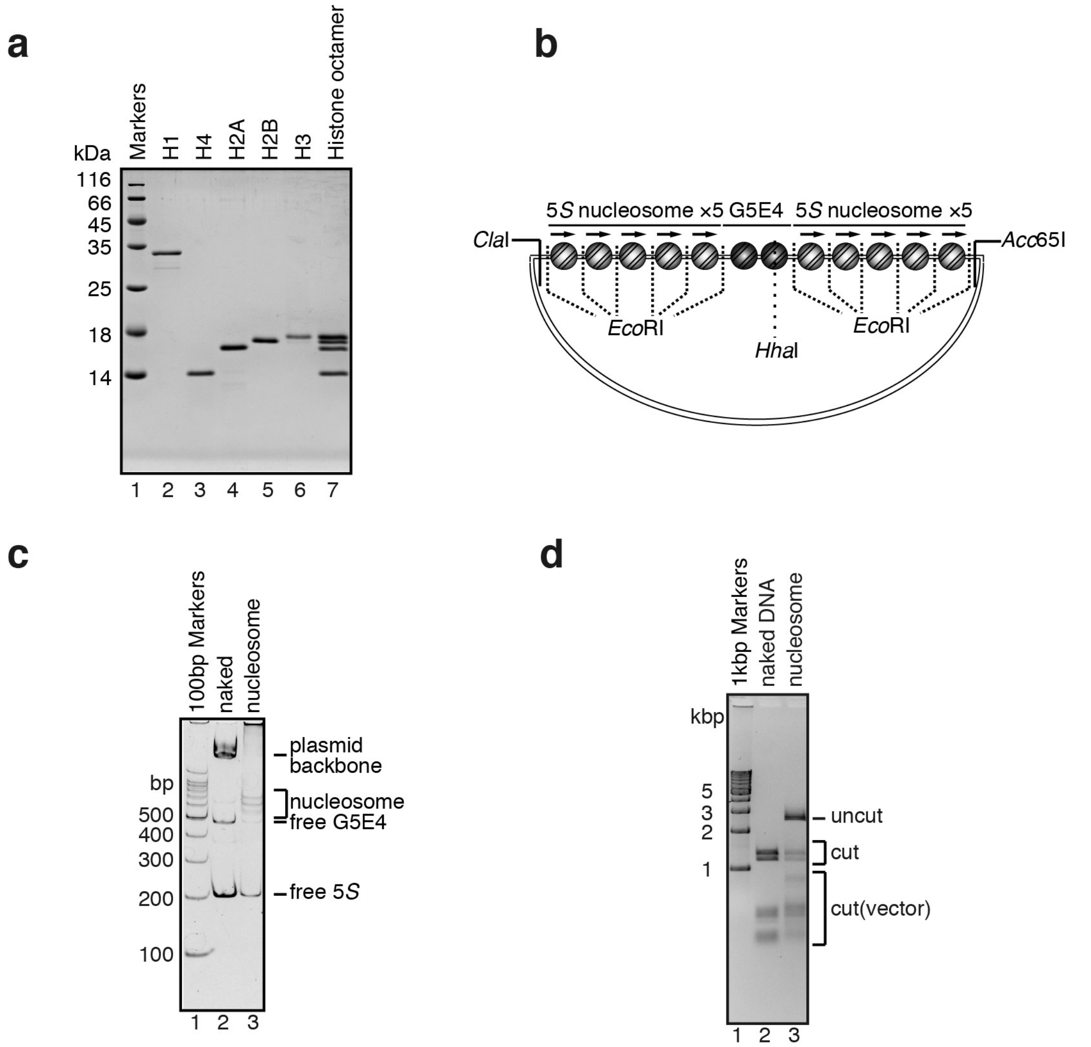

Supplementary Figure S3. Homologous pairing in nucleosome arrays.

(**a**) Human histones and the histone octamer. Purified histones H1, H4, H2A, H2B, and H3.1, and reconstituted histone octamers were analyzed by 18% SDS-PAGE with Coomassie Brilliant Blue staining.

(**b**) Schematic diagram of the plasmid DNA used in the D-loop assay. The arrow indicates the homologous region of the 5*S* ssDNA 90-mer. The *Eco*RI and *Hha*I sites are indicated.

(**c**) *Eco*RI analysis of the nucleosomal plasmid DNA. The reconstituted nucleosome arrays were digested with *Eco*RI, and the products were analyzed by 6% native polyacrylamide gel electrophoresis with EtBr staining. The

nucleosome occupancy on the 5*S* DNA sequence was estimated by comparing the amounts of the nucleosome-free 5*S* DNA fragment with the *Eco*RI-treated naked DNA. Similar results were obtained in at least three independent experiments.

(**d**) *Hha*I analysis of the nucleosomal plasmid DNA. The reconstituted nucleosome arrays were digested with *Hha*I, *Cla*I and *Acc*65I. The products were analyzed by 1.2% agarose gel electrophoresis with EtBr staining. The nucleosome occupancies at the *Hha*I site on the G5E4 sequence were estimated by comparing the amounts of uncut and cut fragments. Similar results were obtained in at least three independent experiments.

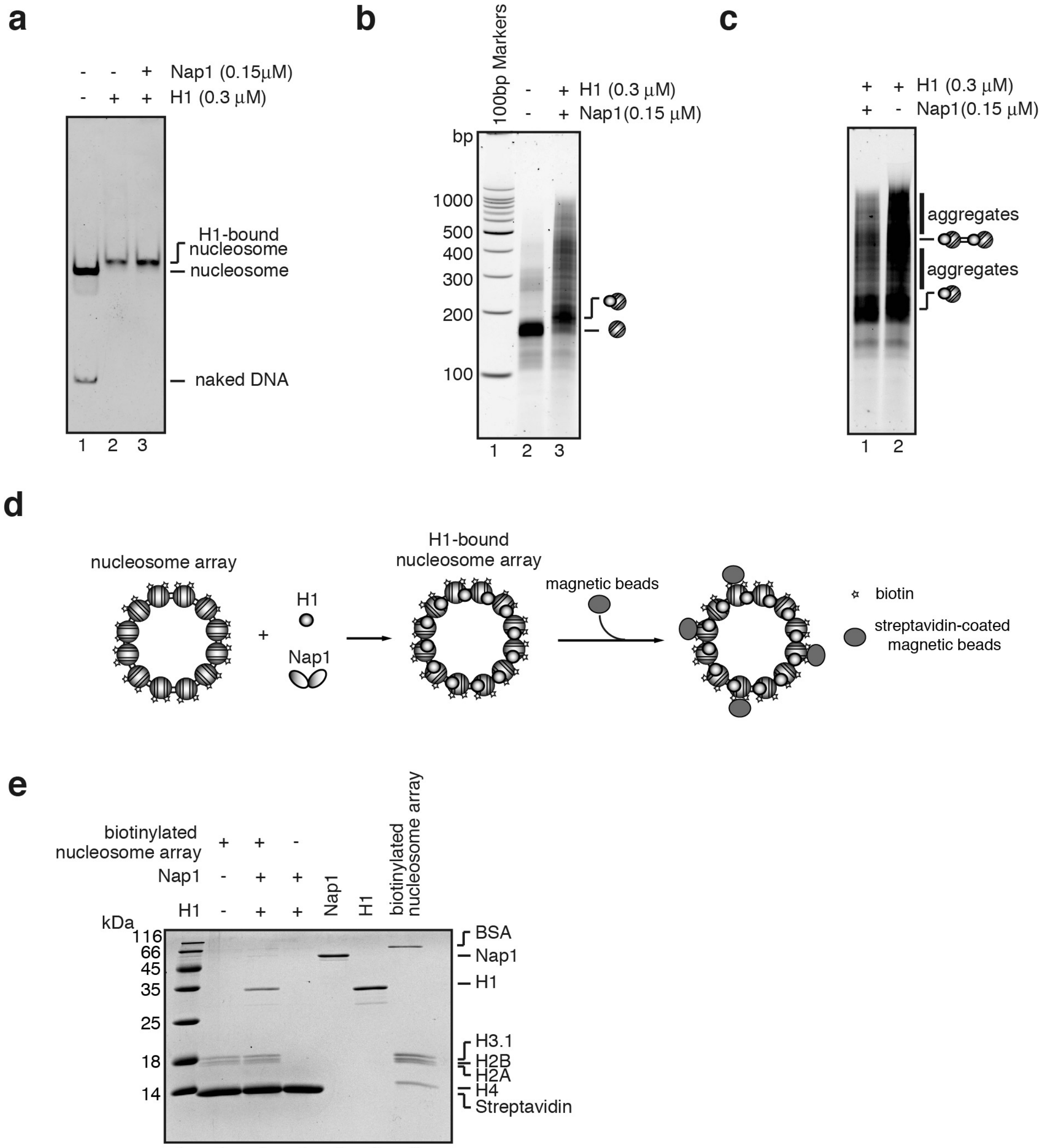

Supplementary Figure S4. Histone H1 binding to nucleosomes and nucleosome arrays with Nap1.

(**a**) Gel-shift assay for histone H1 binding to mono-nucleosomes in the presence of Nap1. Histone H1 (0.3 μM) was mixed with Nap1 (0.15 μM), and the sample was kept on ice for 10 min to form the H1-Nap1 complex. The H1-Nap1 complex was added to nucleosomes (30 μM) reconstituted with the 193 base-pair DNA. After a 25 min incubation at 37°C, the reaction products were analyzed by 5% native polyacrylamide gel electrophoresis in 0.5× TBE buffer

with EtBr staining. Similar results were obtained at least three times, under slightly different experimental conditions.

(**b**) The chromatosome array, assembled with nucleosomes and histone H1 in the presence of Nap1 (Nap1/H1 ratio=0.5), was analyzed by a micrococcal nuclease (MNase) treatment assay. The nucleosome or chromatosome array was digested by MNase, and the DNA fragments were analyzed by native PAGE. Similar results were obtained at least twice.

(**c**) The chromatosome arrays, assembled with nucleosomes and histone H1 in the presence or absence of Nap1, were analyzed by a micrococcal nuclease (MNase, 2 units) treatment assay in 10 µl of reaction buffer, containing 22 mM HEPES-NaOH (pH 7.5), 16 mM Tris-HCl (pH 7.5), 80 mM KCl, 60 mM NaCl, 0.2 mM EDTA, 1 mM DTT, 0.7 mM 2-mercaptoethanol, 20 µM phenylmethylsulfonyl fluoride, 9% glycerol, 1 mM $CaCl_2$, 1 mM $MgCl_2$, and 100 µg/ml BSA. DNA fragments were analyzed by native PAGE. Similar results were obtained at least twice.

(**d**) Schematic diagram of the pull-down assay for evaluating the Nap1-mediated H1 binding to the nucleosome array.

(**e**) The pull-down assay for evaluating the Nap1-mediated H1 binding to the nucleosome array. The biotinylated nucleosome array was reconstituted under the same conditions used for the homologous pairing assay. Histone H1 was assembled on the biotinylated nucleosome array in the presence of Nap1 (Nap1/H1 ratio=0.5), and the nucleosome array was captured by the addition of streptavidin-conjugated magnetic beads. The proteins that copelleted with the magnetic beads were analyzed by SDS–PAGE with Coomassie Brilliant Blue staining.

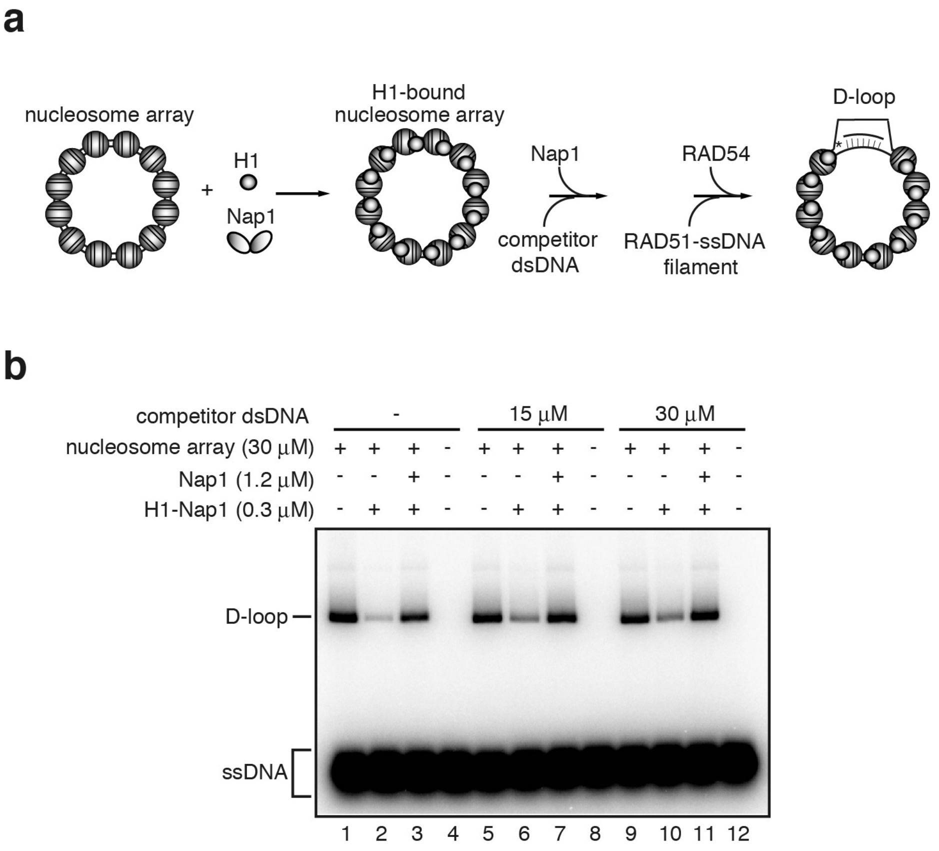

Supplementary Figure S5. Assay for homologous pairing in the presence of a heterologous competitor DNA.

(**a**) Schematic diagram of the homologous-pairing assay in the presence of a heterologous competitor dsDNA.

(**b**) The homologous-pairing assay with a nucleosome array containing histone H1, in the presence or absence of a heterologous competitor dsDNA. Histone H1 (0.3 μM) with Nap1 (Nap1/H1 ratio=0.5) was added to the nucleosome array. After an incubation, the histone chaperone Nap1 and the indicated amounts of the heterologous competitor dsDNA were added, and the reactions were initiated by the addition of the RAD51-ssDNA (90-mer) filament and RAD54. The RAD51-ssDNA filament and RAD54 were added to all lanes.

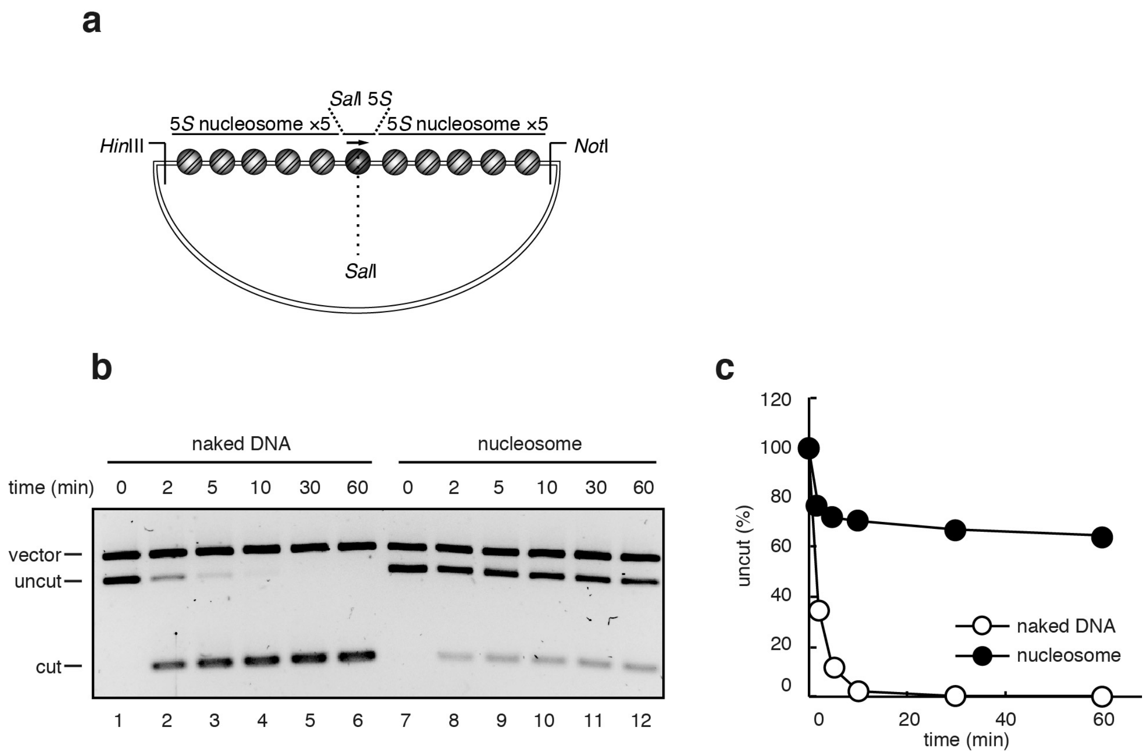

Supplementary Figure S6. Nucleosome occupancy at the *SalI* site of the nucleosome array.

(**a**) Schematic diagram of the plasmid DNA used in the nucleosome remodeling assay. The arrow indicates the homologous region of the *SalI* 5*S* ssDNA 70-mer. The *SalI* site on the *SalI* 5*S* nucleosome is indicated.

(**b**) The *SalI* analysis. The reconstituted nucleosome array or the naked dsDNA was treated with *SalI* for the indicated times in 20 mM HEPES-NaOH buffer (pH 7.5), containing 1 mM Tris-HCl (pH 7.5), 42 mM NaCl, 100 mM KCl, 0.1 mM EDTA, 1 mM DTT, 10% glycerol, 2 mM $MgCl_2$, 1 mM ATP, and 100 µg/ml BSA. Similar results were obtained at least twice for the different time points.

(**c**) Graphic representation of the experiments shown in panel **b**.

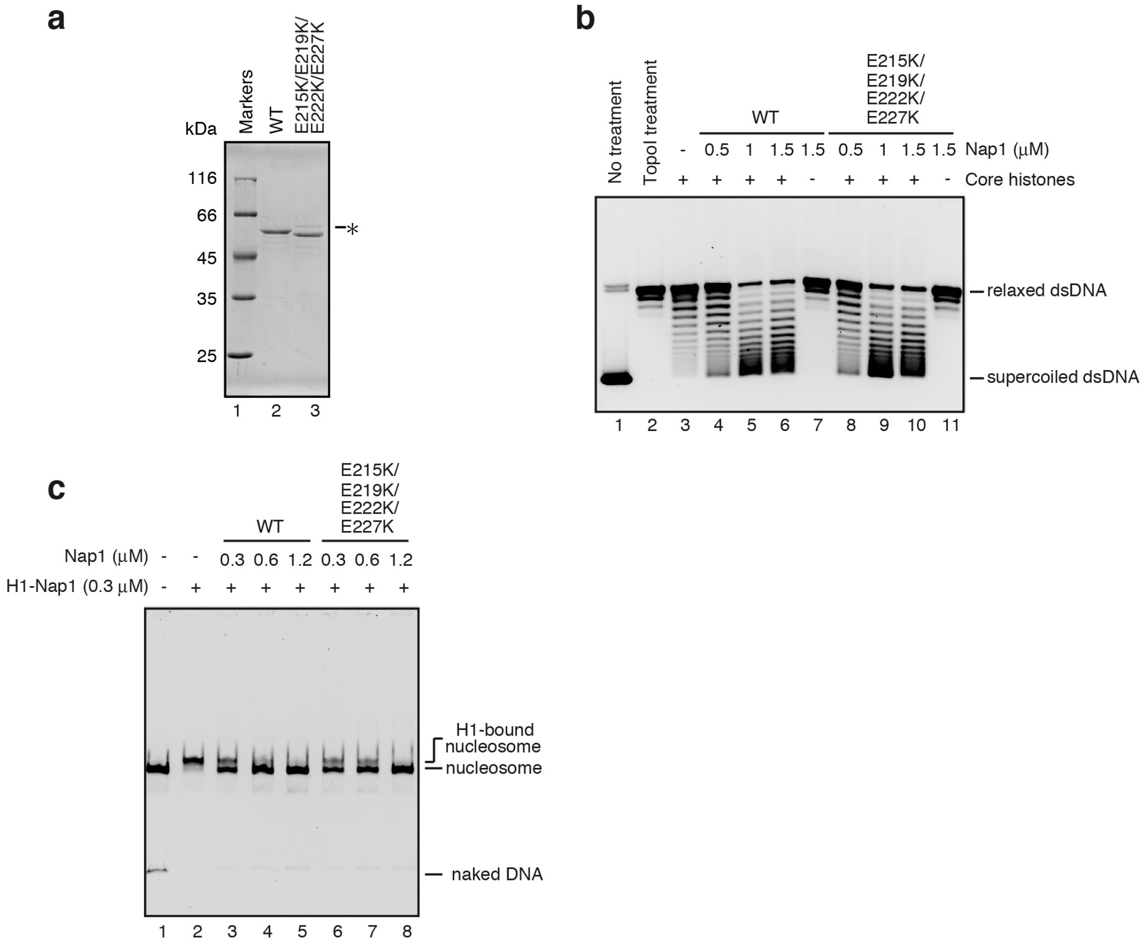

Supplementary Figure S7. Purification and activities of the Nap1(E215,219,222,227K) mutant

(**a**) Purified Nap1 and the Nap1(E215,219,222,227K) mutant were analyzed by 12% SDS-PAGE with Coomassie Brilliant Blue staining. The asterisk indicates the read-through product.

(**b**) Topological assay for nucleosome formation. Nucleosomes were reconstituted on the relaxed plasmid DNA by wild type Nap1 and the Nap1(E215,219,222,227K) mutant in the presence of wheat germ topoisomerase I. Similar results were obtained at least twice.

(**c**) Histone H1 disassembly activities of Nap1 and the Nap1(E215,219,222,227K) mutant. Nap1 and the Nap1(E215,219,222,227K) mutant titration experiments with chromatosomes reconstituted with a 193 base-pair DNA fragment. Histone H1 (0.3 μM) was assembled onto the nucleosome with wild type Nap1 (0.15 μM). Similar results were obtained at least twice.

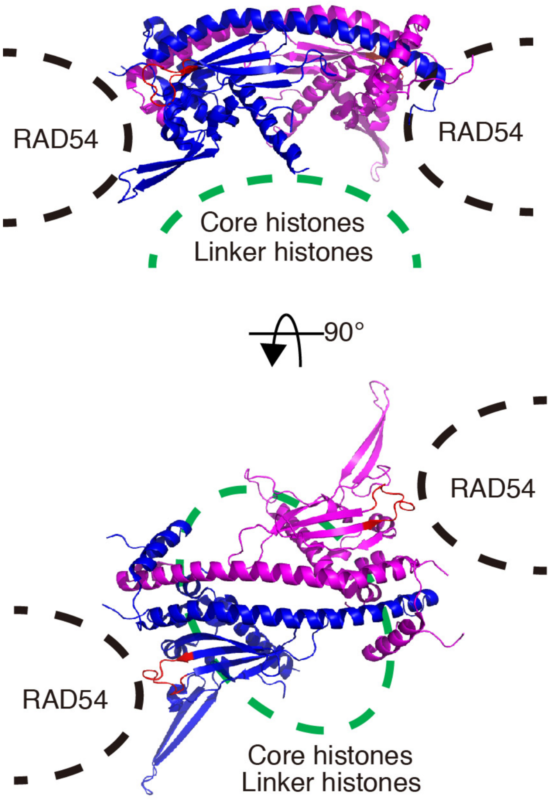

Supplementary Figure S8. Putative RAD54 interaction surfaces on the Nap1 structure.

Four acidic residues, Glu215, Glu219, Glu222, and Glu227 (red), are present on the yeast Nap1 structure (PDB 2Z2R: blue and magenta). These Nap1 glutamate residues are exposed to the solvent, and potentially interact with basic proteins, such as RAD54 (ellipses with dashed black lines). The previously reported binding site for core and linker histones is indicated in the ellipse with a dashed green line[23, Supplementary ref. 60].

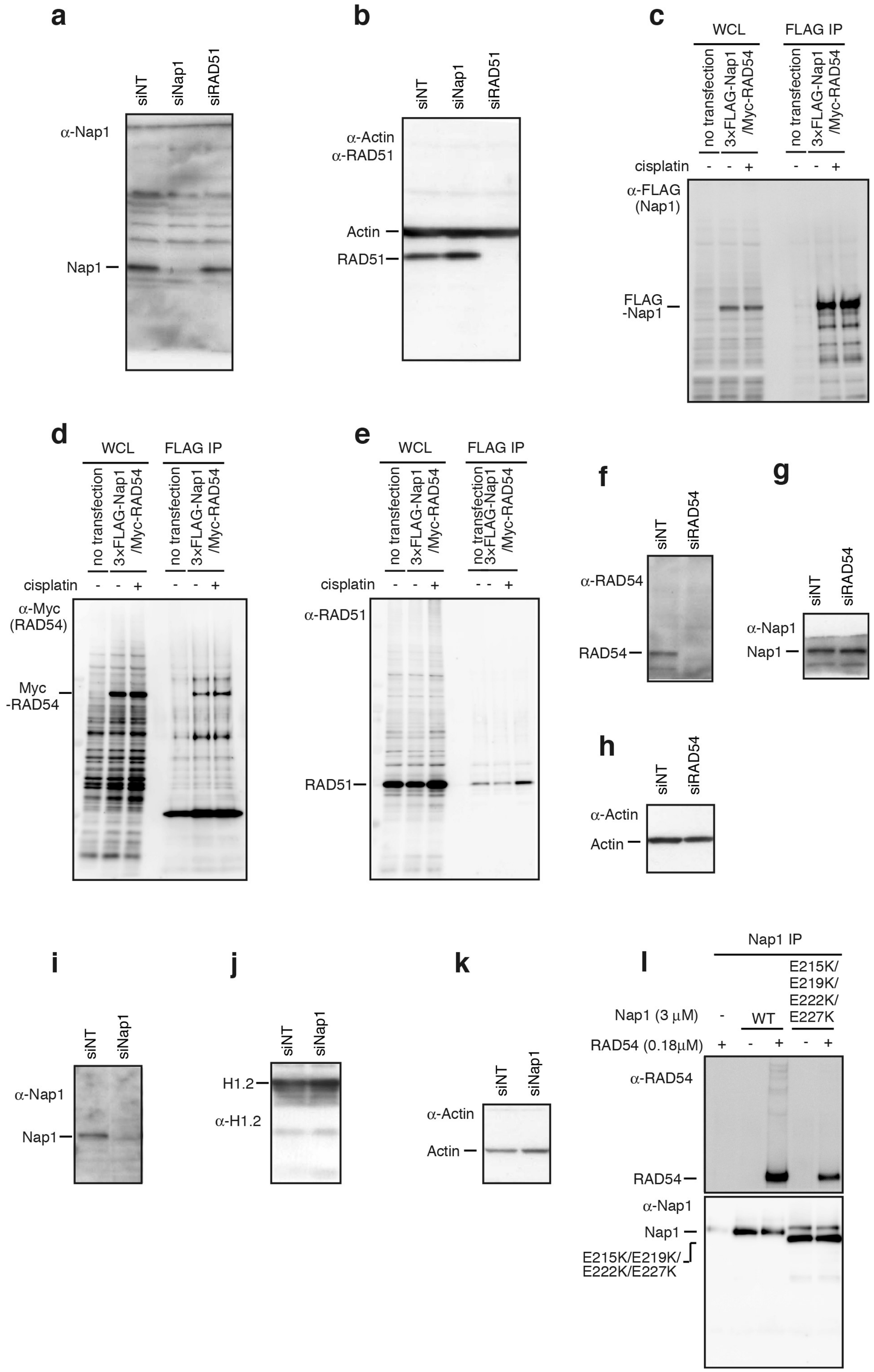

a
siNT
siNap1
siRAD51
α-Nap1
Nap1
b
siNT
siNap1
siRAD51
α-Actin
α-RAD51
Actin
RAD51
c
WCL
FLAG IP
no transfection
3×FLAG-Nap1 /Myc-RAD54
cisplatin - - + - - +
α-FLAG (Nap1)
FLAG -Nap1
d
WCL
FLAG IP
no transfection
3×FLAG-Nap1 /Myc-RAD54
cisplatin - - + - - +
α-Myc (RAD54)
Myc -RAD54
e
WCL
FLAG IP
no transfection
3×FLAG-Nap1 /Myc-RAD54
cisplatin - - + - - +
α-RAD51
RAD51
f
siNT
siRAD54
α-RAD54
RAD54
g
siNT
siRAD54
α-Nap1
Nap1
h
siNT
siRAD54
α-Actin
Actin
i
siNT
siNap1
α-Nap1
Nap1
j
siNT
siNap1
H1.2
α-H1.2
k
siNT
siNap1
α-Actin
Actin
l
Nap1 IP
Nap1 (3 μM) - WT E215K/ E219K/ E222K/ E227K
RAD54 (0.18μM) + - + - +
α-RAD54
RAD54
α-Nap1
Nap1
E215K/E219K/ E222K/E227K

Supplementary Figure S9. Full-length images for Figure 1d, 2a, 2c, 5d and 7b

(**a**) Nap1 (Fig. 1d). (**b**) RAD51 and Actin. The anti-rabbit RAD51 polyclonal antibody and the anti-mouse Actin monoclonal antibody were mixed, and were used for western blotting detection with anti-rabbit and anti-mouse secondary antibodies (Fig. 1d). (**c**) Flag-Nap1 (Fig. 2a). (**d**) Myc-RAD54 (Fig. 2a). (**e**) RAD51 (Fig. 2a). (**f**) RAD54 (Fig. 2c). (**g**) Nap1 (Fig. 2c). (**h**) Actin (Fig. 2c). (**i**) Nap1 (Fig. 5d). (**j**) H1.2 (Fig. 5d). (**k**) Actin (Fig. 5d). (**l**) RAD54, Nap1, and the Nap1(E215,219,222,227K) mutant (Fig. 7b).

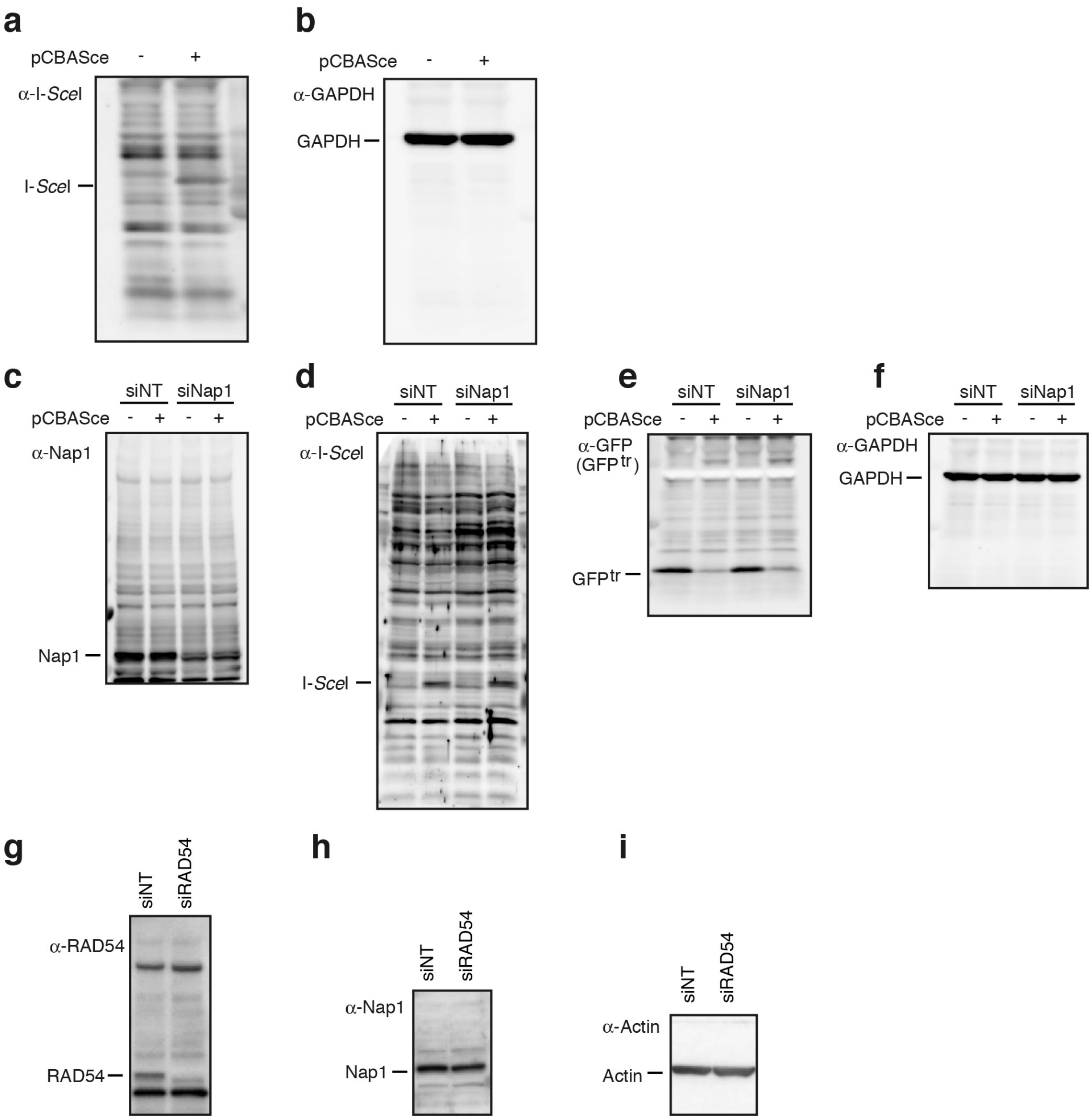

Supplementary Figure S10. Full-length images for Supplementary Figure S1 and S2a

(**a**) I-*Sce*I (supplementary Fig. S1b). (**b**) GAPDH (supplementary Fig. S1b). (**c**) Nap1 (supplementary Fig. S1c). (**d**) I-*Sce*I (supplementary Fig. S1c). (**e**) GFP^{tr} (supplementary Fig. S1c). (**f**) GAPDH (supplementary Fig. S1c). (**g**) RAD54 (supplementary Fig. S2a). (**h**) Nap1 (supplementary Fig. S2a). (**i**) Actin (supplementary Fig. S2a).

Supplementary Table

Supplementary Table S1. DNA sequences of the oligonucleotides.

	Name	Sequence
1	ssDNA 90-mer containing 5*S* DNA sequence	CCGGTATATTCAGCATGGTATGGTCGTAGG CTCTTGCTTGATGAAAGTTAAGCTATTTAA AGGGTCAGGGATGTTATGACGTCATCGGCT
2	ssDNA 70-mer containing *Sal*I 5*S* DNA sequence	GGTGCATTCACCCTGGTATAGTCGACGTCT CTTGCTTGATGAAAGTTAAGCTATTTAAAG GGTCAGGGAT

Supplementary Methods

Purification of human histone H1.2

The human histone H1.2 cDNA was inserted into the *Nde*I-*Bam*HI sites of the pET21a vector, as the N-terminally SUMO-fused protein. The His_6-SUMO-tagged H1.2 protein was produced in the *Escherichia coli* BL21 (DE3) strain, which carried the minor tRNA expression vector (Codon(+)RIL; Stratagene). The cells producing His_6-SUMO-tagged H1.2 were resuspended in buffer A (50 mM Tris-HCl (pH 8.0), 2 mM 2-mercaptoethanol, 500 mM NaCl, and 10% glycerol) and disrupted by sonication. The cell lysate was clarified by centrifugation, and the supernatant was gently mixed with Ni-NTA agarose beads (Qiagen) for 15 min at 4°C. The resin was packed in a column and washed with buffer B (50 mM Tris-HCl (pH 7.5), 500 mM NaCl, 2 mM 2-mercaptoethanol, 5 mM imidazole, and 10% glycerol), and the protein was eluted by a linear gradient of 5 to 500 mM imidazole. The peak fractions containing His_6-SUMO-tagged H1.2 were collected, and the His_6-SUMO segment was removed by PreScission Protease (5 unit/mg, GE Healthcare) treatment during dialysis against buffer C (20 mM Tris-HCl (pH 7.5), 100 mM NaCl, 2 mM 2-mercaptoethanol, and 10% glycerol). After the removal of the His_6-SUMO segment, the sample was subjected to MonoS (GE Healthcare) column chromatography. The column was washed with buffer D (20 mM Tris-HCl (pH 7.5), 2 mM 2-mercaptoethanol, 350 mM NaCl, and 10% glycerol), and

H1.2 was eluted by a linear gradient of 350 - 2,000 mM NaCl. The purified H1.2 was stored at -80°C in buffer C.

Preparation of human histones, Nap1, and sNASP

Human histones H2A, H2B, H3.1, and H4 were produced in *Escherichia coli* cells, and were purified by the method described previously[31]. Human Nap1[46], and sNASP[53] were overexpressed in *Escherichia coli* cells, and were purified. For the purification of the human Nap1(E215K,E219K,E222K,E227K) mutant, the flow-through fractions from the Heparin Sepharose column (GE Healthcare) were collected, and the sample was further purified by the method described previously[46].

Preparation of human RAD51 and RAD54

Human RAD51 was overexpressed in *Escherichia coli* cells, and was purified as described previously[54]. The human RAD54 cDNA was inserted into the pFastBac HTc vector (Life Technologies), and the recombinant human RAD54 baculovirus was generated[55]. Sf9 insect cells infected with the human RAD54 baculovirus were harvested after 48 h. The cells were disrupted by sonication in buffer A (50 mM Tris-HCl (pH 7.8), 10 mM imidazole, 300 mM KCl, 2 mM 2-mercaptoethanol, 10% glycerol, and 0.1× protease inhibitor cocktail (Nacalai Tesque). The cell lysate was centrifuged, and the supernatant was gently mixed with Ni-NTA agarose beads (Qiagen) for 45 min at 4°C. The resin was packed

in a column and washed with buffer A. The protein was eluted by a linear gradient of 10 to 300 mM imidazole. The peak fractions containing His_6-tagged RAD54 were collected, and dialyzed against buffer B (50 mM Tris-HCl (pH 7.8), 10% glycerol, 100 mM KCl, 0.5 mM EDTA, and 2 mM 2-mercaptoethanol). The sample was loaded on a Heparin Sepharose column (GE Healthcare), which was equilibrated with buffer B. The resin was washed with buffer B, and the protein was eluted by a linear gradient of 100 to 1,000 mM KCl. The peak fractions containing His_6-tagged RAD54 were collected, and the His_6-tag was removed by TurboTEV Protease (70 unit/mg, Accelagen) treatment during dialysis against buffer C (20 mM Tris-HCl (pH 7.5), 1 mM EDTA, 10% glycerol, 200 mM KCl, and 2 mM 2-mercaptoethanol). After the removal of the His_6-tag, the sample was loaded on a Q Sepharose column (GE Healthcare). The flow-through fractions were collected, and the sample was diluted with buffer D (20 mM Tris-HCl (pH 7.5), 10% glycerol, and 2 mM 2-mercaptoethanol). The proteins were loaded on an SP Sepharose column (GE Healthcare), and the resin was washed with buffer E (20 mM Tris-HCl (pH 7.5), 10% glycerol, 100 mM KCl, and 2 mM 2-mercaptoethanol). RAD54 was eluted with a linear gradient of 100-600 mM KCl. The peak fractions containing RAD54 were dialyzed against buffer F (20 mM potassium phosphate (pH 7.5), 10% glycerol, 200 mM KCl, and 2 mM 2-mercaptoethanol), and were subjected to chromatography on a Hydroxyapatite column (Bio-Rad). The column was washed with buffer F, and RAD54 was eluted with a linear gradient of 20-300

mM potassium phosphate. The purified RAD54 was stored at -80°C in buffer G (20 mM Tris-HCl (pH 7.5), 30% glycerol, 400 mM KCl, and 2 mM 2-mercaptoethanol).

Assembly of nucleosome arrays and nucleosomes

Nucleosome arrays were reconstituted on the plasmid DNAs by the salt dialysis method. Nucleosome arrays were reconstituted at either an R value of 1 (assay for homologous pairing) or an R value of 1.2 (assay for nucleosome remodeling) for the ratio of histone octamers per 200 base pairs of donor DNA[9]. For the nucleosome arrays used in the D-loop formation and topological assays, negatively supercoiled DNA was used for the nucleosome array reconstitution, because it is required for efficient detection of the reaction products in these assays. Nucleosomes were prepared by the method described previously[58]. For the nucleosome reconstitution with the 193-base-pair DNA fragment (100 µg), the purified histone octamer (106 µg) containing human histones H2A, H2B, H3.1, and H4 was used.

Assay for H1 binding to nucleosomes

Histone H1 (300 nM) was mixed with Nap1 (150 nM), and the sample was kept on ice for 10 min to allow H1-Nap1 complex formation. The H1-Nap1 complex was added to nucleosomes reconstituted with the 193 base-pair DNA (30 µM) to form chromatosomes, in a reaction buffer containing 22 mM HEPES-NaOH

(pH 7.5), 9 mM Tris-HCl (pH 7.5), 60 mM KCl, 43 mM NaCl, 0.2 mM EDTA, 1 mM DTT, 0.6 mM 2-mercaptoethanol, 15 µM phenylmethylsulfonyl fluoride, 7.5% glycerol, 1 mM $MgCl_2$, 1 mM $CaCl_2$, and 100 µg/ml BSA. After a 15 min incubation at 37°C, Nap1 or Nap1 storage buffer was added to the reaction mixture, and the histone H1-eviction reactions were initiated. After a 10 min incubation at 37°C, the reaction products were analyzed by 5% native polyacrylamide gel electrophoresis in 0.5x TBE buffer with EtBr staining.

MNase assay

Histone H1 (300 nM) was mixed with Nap1 (150 nM), and the sample was kept on ice for 10 min to form the H1-Nap1 complex. The H1-Nap1 complex was added to nucleosomal dsDNA (30 µM) to form chromatosomes. After a 10 min incubation at 37°C, the nucleosomes or chromatosomes were treated with 2 units of MNase (Takara), in 10 µl of reaction buffer, containing 22 mM HEPES-NaOH (pH 7.5), 14 mM Tris-HCl (pH 7.5), 80 mM KCl, 48 mM NaCl, 0.2 mM EDTA, 1 mM DTT, 0.6 mM 2-mercaptoethanol, 15 µM phenylmethylsulfonyl fluoride, 8% glycerol, 1 mM $MgCl_2$, 2 mM $CaCl_2$, and 100 µg/ml BSA. After a 10 min incubation at room temperature, the reactions were stopped by the addition of 2 µl of stop solution, containing SDS (0.2%) and proteinase K (1.4 mg/ml, Roche Applied Science), and incubated further for 15 min. The reactions were extracted with phenol/chloroform, and the DNA was precipitated with ethanol. The samples were then fractionated by 6% native polyacrylamide gel

electrophoresis in 0.2x TBE buffer. The DNA was visualized by EtBr staining.

Assay for protein-protein interactions *in vitro*

Purified Nap1 (100 µg) was covalently conjugated to Affi-Gel 15 beads (100 µl, Bio-Rad), in a reaction buffer containing 20 mM HEPES-NaOH (pH 7.5), 150 mM NaCl, 0.5 mM EDTA, 1 mM DTT, 0.1 mM phenylmethylsulfonyl fluoride, and 10% glycerol, according to the manufacturer's instructions. To block the remaining active ester sites, ethanolamine (100 mM, pH 8.0) was added, and the beads were incubated at 4°C for 1 hr. To remove the unbound proteins, the beads were washed three times with reaction buffer containing 0.05% Triton X-100. After washing the beads, the Affi-Gel 15-protein matrices were adjusted to 50% slurries, and stored at 4°C. The control beads were prepared by the same method in the absence of Nap1. For the RAD51- and RAD54-binding assays, the Nap1 beads (7.5 µl) were incubated with RAD51 (20 µg) or RAD54 (20 µg) in 65 µl of reaction buffer, containing 6 mM HEPES-NaOH (pH 7.5), 14 mM Tris-HCl (pH 7.5), 110 mM KCl, 100 mM NaCl, 0.2 mM EDTA, 0.4 mM DTT, 1 mM 2-mercaptoethanol, 39 µM phenylmethylsulfonyl fluoride, 15% glycerol, and 0.3% Triton X-100. After a 2.5 hr incubation at room temperature, the beads were washed twice with wash buffer, containing 6 mM HEPES-NaOH (pH 7.5), 13 mM Tris-HCl (pH 7.5), 130 mM KCl, 100 mM NaCl, 0.2 mM EDTA, 0.3 mM DTT, 1 mM 2-mercaptoethanol, 33 µM phenylmethylsulfonyl fluoride, 17% glycerol, and 0.3% Triton X-100. For the RAD51-RAD54-binding assay,

the Nap1 beads (7.5 µl) were initially incubated with RAD54 (20 µg). After a 1.5 hr incubation, RAD51 (20 µg) was added to the reaction mixture containing RAD54 and the Nap1 beads, and the mixture was incubated further for 1 hr. The proteins that copelleted with the Nap1 beads were denatured, and analyzed by 12% SDS-polyacrylamide gel electrophoresis with Coomassie Brilliant Blue staining.

For the gel mobility shift assay, Nap1 (6 µM) was mixed with RAD54 (3 or 6 µM) in 10 µl of 12 mM Tris-HCl buffer (pH 7.5), containing 45 mM NaCl, 120 mM KCl, 0.2 mM EDTA, 1 mM DTT, 0.6 mM 2-mercaptoethanol, 30 µM phenylmethylsulfonyl fluoride, and 12% glycerol. After an incubation for 30 min at 37°C, the Nap1-RAD54 complexes were analyzed by 5% native polyacrylamide gel electrophoresis with Coomassie Brilliant Blue staining.

For the pull-down assays with Nap1 and the Nap1(E215K,E219K,E222K,E227K) mutant, each purified protein (3 µM) was mixed with RAD54 (0.18 µM) in 10 µl of 12 mM Tris-HCl buffer (pH 7.5), containing 120 mM KCl, 45 mM NaCl, 0.2 mM EDTA, 1 mM DTT, 0.6 mM 2-mercaptoethanol, 30 µM phenylmethylsulfonyl fluoride, and 12% glycerol. After an incubation for 30 min at 37°C, 200 µl of anti-Nap1 polyclonal antibody (5 µg/ml) was added to the reaction mixture. After an incubation for 30 min at room temperature, 10 µl of Dynabeads protein G (Invitrogen) was added to the reaction mixture, which was incubated further for 30 min at room temperature. The beads were washed three times with wash buffer, containing 6 mM

HEPES-NaOH (pH 7.5), 13 mM Tris-HCl (pH 7.5), 130 mM KCl, 100 mM NaCl, 0.2 mM EDTA, 0.3 mM DTT, 1 mM 2-mercaptoethanol, 33 µM phenylmethylsulfonyl fluoride, 17% glycerol, and 0.3% Triton X-100. The proteins that copelleted with the Dynabeads were denatured, and were separated by 5-20% gradient SDS–PAGE. RAD54, Nap1, and the Nap1(E215K,E219K,E222K,E227K) mutant were detected by western blotting with an anti-RAD54 polyclonal antibody and an anti-Nap1 polyclonal antibody, respectively.

Assay for protein-protein interactions *in vivo*

HEK293T cells were cultured and maintained at 37°C (5% CO_2) in Dulbecco's modified Eagle's medium (Nacalai Tesque), supplemented with 10% fetal calf serum and penicillin-streptomycin. HEK293T cells were transiently transfected with the FLAG(3x)-Nap1 plasmid and the Myc-RAD54 plasmid, using GeneJuice (Novagen). For the induction of DNA damage, HEK293T cells were treated with cisplatin (10 µM) for 24 hrs. Subsequently, the cells were suspended in 150 mM KCl buffer, containing 20 mM Tris-HCl (pH 7.5), 2 mM EDTA, 0.2 mM 2-mercaptoethanol, 10% glycerol, 0.1% Tween-20, and 1x protease inhibitor cocktail (Nacalai Tesque). The cells were disrupted by freezing in liquid nitrogen and thawing, and the samples were sonicated to solubilize the chromosomal proteins. After the lysate (0.8 mg of protein) was incubated with anti-FLAG-antibody-conjugated agarose (20 µl, Sigma) at 4°C

for 4 hrs, the beads were washed three times with 150 mM KCl buffer. The proteins that copelleted with the FLAG-antibody-conjugated agarose were eluted with 3x FLAG peptide (15 µg, Sigma). The whole-cell lysate (WCL, 3.8 µg of protein) and the eluted fractions were separated by 5-20% gradient SDS–PAGE. The FLAG(3x)-tagged Nap1, Myc-tagged RAD54, endogenous RAD51, and endogenous histone H3 were detected by western blotting with an anti-FLAG monoclonal antibody (Sigma), an anti-Myc monoclonal antibody (Sigma), an anti-RAD51 polyclonal antibody (but not for RAD51 paralogs, such as RAD51B, RAD51C, RAD51D, XRCC2, and XRCC3), and an anti-histone H3 polyclonal antibody (Cell Signaling), respectively.

Accumulation of γH2AX after laser UVA microirradiation

For the detection of γH2AX, GM0637 cells were washed with cold PBS at 10 minutes after laser UVA microirradiation, and were then incubated for 5 min at room temperature with CSK buffer containing 0.5% Triton. Thereafter, the cells were fixed with PBS containing 4% paraformaldehyde for 10 minutes, and were then incubated with mouse anti- γH2AX antibodies (1:40,000, Upstate Biotechnology) in PBS containing 1% BSA at 37°C for 30 min. A Cy3-conjugated sheep anti-mouse antibody (1:1,000; Jackson ImmunoResearch) was used as a secondary antibody. Nuclei were stained with DAPI. Samples were examined with a ZEISS Axioplan2 microscope controlled by Axiovision.

Topological assay for nucleosome formation

The H3/H4 complex (8.5 ng) and the H2A/H2B complex (8.5 ng) were pre-incubated with Nap1 at 37°C for 15 min in 30 mM Tris-HCl buffer (pH 7.5), containing 0.6 mM EDTA, 170 mM NaCl, 3 mM DTT, 0.5 mM phenylmethylsulfonyl fluoride, 7% glycerol, and 2 mM $MgCl_2$. The nucleosome assembly reaction was initiated by the addition of relaxed circular ϕX174 dsDNA (100 ng), which had been pre-incubated with 1.3 U wheat germ topoisomerase I (Promega) at 37°C for 150 min in 20 mM Tris-HCl buffer (pH 7.5), containing 84 mM NaCl, 5 mM dithiothreitol, 0.2 mM EDTA, 8% glycerol, and 2 mM $MgCl_2$. After a 60 min incubation at 37°C, the reactions were terminated by the addition of 60 µL of a proteinase K solution, containing 20 mM Tris-HCl (pH 8.0), 0.5% SDS, 20 mM EDTA, and 0.5 mg/mL proteinase K (Roche Applied Science). After a 15 min incubation at 37°C, the reactions were extracted with phenol/chloroform, precipitated with ethanol, and then analyzed by 1% agarose gel electrophoresis. The DNA products were visualized by SYBR Gold (Invitrogen) staining.

Pull-down assay with biotinylated nucleosome array

Histone octamers were reconstituted with recombinant histones H2A(A113C), H2B, H3(C110A), and H4, as described previously[31]. Biotinylated histone octamers were prepared as described[9]. Nucleosome arrays were reconstituted with the ratio of one histone octamer per 200 base pairs of dsDNA[9]. The H1-

bound biotinylated nucleosome array was reconstituted under the same conditions as the H1-nucleosome array reconstitution step in the homologous pairing assay. The H1-bound biotinylated nucleosome array (1.8 ng of dsDNA) was then captured by the addition of 0.4 mg of streptavidin-conjugated magnetic beads (Dynabeads M-280 Streptavidin, Invitrogen) that had been preblocked in reaction buffer, containing 0.1 mg/ml BSA and 0.2 mg/ml sonicated salmon sperm DNA. After a 10 min incubation, the magnetic beads were washed three times with reconstitution buffer for the H1-bound biotinylated nucleosome array, containing 0.01% NP-40. The proteins that copelleted with the magnetic beads were denatured, and were analyzed by SDS–PAGE with Coomassie Brilliant Blue staining.

Supplementary Reference

60. D'Arcy, S. *et al.* Chaperone Nap1 shields histone surfaces used in a nucleosome and can put H2A-H2B in an unconventional tetrameric form. *Mol. Cell. 51,* 662-677 (2013).
